# Minimally invasive pancreaticoduodenectomy for periampullary disease: a comprehensive review of literature and meta-analysis of outcomes compared with open surgery

**DOI:** 10.1186/s12876-017-0691-9

**Published:** 2017-11-23

**Authors:** Ke Chen, Yu Pan, Xiao-long Liu, Guang-yi Jiang, Di Wu, Hendi Maher, Xiu-jun Cai

**Affiliations:** 1 Department of General Surgery, Sir Run Run Shaw Hospital, School of Medicine, Zhejiang University, 3 East Qingchun Road, Hangzhou, Zhejiang Province 310016 China; 2 School of Medicine, Zhejiang University, 866 Yuhangtang Road, Hangzhou, Zhejiang Province 310058 China

**Keywords:** Laparoscopy, Robot, Minimally invasive, Pancreaticoduodenectomy, Morbidity, Review, Meta-analysis

## Abstract

**Background:**

Minimally invasive pancreatoduodenectomy (MIPD) has been gradually attempted. However, whether MIPD is superior, equal or inferior to its conventional open pancreatoduodenectomy (OPD) is not clear.

**Methods:**

Studies published up to May 2017 were searched in PubMed, Embase, Cochrane Library, and Web of Science. Main outcomes were comprehensively reviewed and measured including conversion to open approach, operation time (OP), estimated blood loss (EBL), transfusion, length of hospital stay (LOS), overall complications, postoperative pancreatic fistula (POPF), delayed gastric emptying (DGE), post-pancreatectomy hemorrhage (PPH), readmission, reoperation and reasons of preoperative death, number of retrieved lymph nodes (RLN), surgical margins, recurrence, and survival. The software of Review Manage version 5.1 was used for meta-analysis.

**Results:**

One hundred studies were included for systematic review and 26 out of them (totally 3402 cases, 1064 for MIPD, 2338 for OPD) were included for meta-analysis. In the early years, most articles were case reports or non-control case series studies, while in the last 6 years high-volume and comparative researches were increasing gradually. Systematic review revealed conversion rates of MIPD to OPD ranged from 0% to 40%. The mean or median OP of MIPD ranged from 276 to 657 min. The total POPF rates vary between 3.8% and 50% observed in all systematic reviewed studies. Meta-analysis demonstrated MIPD had longer OP (WMD = 99.4 min; 95%CI: 46.0 ~ 152.8, P < 0.01), lower blood loss (WMD = −0.54 ml; 95% CI, −0.88 ~ −0.20 ml; *P* < 0.01), lower transfusion rate (RR = 0.73, 95%CI: 0.57 ~ 0.94, P = 0.02), shorter LOS (WMD = −3.49 days; 95%CI: -4.83 ~ −2.15, *P* < 0.01). There was no significant difference in time to oral intake, postoperative complications, POPF, reoperation, readmission, perioperative mortality and number of retrieved lymph nodes.

**Conclusion:**

Our study demonstrates MIPD is technically feasible and safety on the basis of historical studies. MIPD is associated with less blood loss, faster postoperative recovery, shorter length of hospitalization and longer operation time. These findings are waiting for being confirmed with robust prospective comparative studies and randomized clinical trials.

**Electronic supplementary material:**

The online version of this article (10.1186/s12876-017-0691-9) contains supplementary material, which is available to authorized users.

## Background

Gagner & Pomp [1] described the first laparoscopic pancreatoduodenectomy (LPD) in 1994. There are two commonly used version of LPD. Laparoscopic-assisted PD (LAPD), also known as hybrid laparoscopic PD (HLPD), is characterized as performing lymphadenectomy and bloc extraction of the specimen under laparoscopy, then requiring an auxiliary incision to finish reconstruction of the digestive tract. If the operation were completely performed intracorporeally, the technique was referred as totally laparoscopic pancreatoduodenectomy (TLPD). Robotic surgery is an emerging technology. If robotic system, usually da Vinci, was used to facilitate procedures of resection or reconstruction, it was referred as RPD. LPD and PPD are collectively known as minimally invasive PD (MIPD). MIPD is technically demanding and postoperative pancreatic fistula or bile leakage can be deadly without secure completion of the complicated pancreatic and biliary anastomoses [2, 3]. Very few institutions have committed to advocating the development of MIPD initially. Nowadays, with recent advances of laparoscopic surgical instruments and the accumulation of operative experience, especially the emerging of robotic system to facilitate the complexity of reconstruction, increasing centers attempted to carry out MIPD for benign and malignant periampullary disease [2, 4]. In fact, during the past six years, there had been a steep increase in the use of MIPD. However, reported series on MIPD were influenced by small sample size, single center experience and selection bias [2]. The majority of them were nonrandomized retrospective trials, while case series and high-quality comparative studies remained limited [2]. Therefore, in the current study, we aimed to perform a comprehensive systematic review of all available studies to improve on these single-institution series as well as a meta-analysis comparing MIPD and conventional open PD (OPD) to evaluate the safety, feasibility, and efficacy of MIPD.

## Methods

### Systematic literature search

Systematic searches of PubMed, Embase, Cochrane Library, and Web of Science were performed to identify articles published up to May 2017. Strategies included the terms “minimally invasive”, “laparoscopic”, “laparoscopy”, “robot”, “robotic”, “Da Vinci”, “pancreaticoduodenectomy”, “Whipple”, “PD” and “pancreatic resection” and “pancreatic surgery”. The language of the publications was confined to English. References were searched for studies not found through the initial search.

### Eligibility criteria

The inclusion criteria for systematic review were prospective or retrospective case series studies assessing surgical outcomes of MIPD, and comparative studies of MIPD and OPD, but case reports were excluded. Abstracts, letters, comments, conference proceedings, reviews were also excluded. Studies included more than 20 cases of MIPD were used for summarizing perioperative the relevant information. Studies included in the meta-analysis fulfilled the following criteria: (1) compared the outcomes of MIPD and OPD; (2) reported on at least one of the outcomes of interest mentioned below; (3) had adequate raw data reported such that we could extract the number or percentage of events for categorical outcomes; (4) continuous variables should both present means and standard deviations (SDs). We did not estimate the means and SDs from medians and ranges or inter-quartile ranges as the method of Hozo et al. [5], because this method may lead to deviation, especially when the sample size is small or the samples exhibit serious skewedness; (5) other kind of pancreatectomy such as enucleation, distal, central or total pancreatectomy; (6) if there was overlap between authors or centers, the higher quality or more recent literature were selected.

### Data extraction and quality assessment

First, two investigators (Chen K and Pan Y) independently tabulated the data. A double-check procedure was performed to make sure of the accuracy of the data extracted. Then, a manager inputted the extracted data into a spreadsheet. The measured outcomes of all eligible publications can be divided into four categories: ① Study baseline: author, geographical region, facility, journal, publishing year, study period, sample volume, study subject, indications and surgical procedure; ② Intraoperative effects: conversion to open approach, operation time (OP), estimated blood loss (EBL), transfusion; ③ Postoperative recovery: time to first flatus or restart oral intake, length of hospital stay (LOS), morbidity and mortality [overall complications, postoperative pancreatic fistula (POPF), delayed gastric emptying (DGE), post-pancreatectomy hemorrhage (PPH), readmission, reoperation and reasons of preoperative death]; ④ Oncologic outcomes: retrieved lymph nodes (RLN), surgical margins, recurrence, and survival. Relied on the complication reporting system of Memorial Sloan-Kettering Cancer Center [6], postoperative complications are categorized into medical complications (pulmonary embolism; nonsurgical infections; respiratory, cardiovascular, or metabolic events; arrhythmia, cerebral vascular accident; and deep venous thrombosis, phlebitis) or surgical complications (pancreatitis, bleeding events, any complication which required reoperation, any anastomotic leakage or stricture, wound complications, intra-abdominal collections, delayed gastric emptying and ileus). The use of intention-to-treat (ITT) analysis was investigated. POPF, DGE, and PPH were diagnosed in accordance with the International Study Group for Pancreatic Fistula (ISGPF) criteria [7–9]. Severe POPF was defined as ISGPF grade B/C [7]. Perioperative mortality was defined as deaths from any cause postoperatively. The Newcastle-Ottawa Quality Assessment Scale (NOS) was utilized to evaluate the quality of the researches included. The scale changes from 0 to 9 stars: researches with a score higher than or equal to 6 could be deemed as good methodologically.

### Statistical analysis

The risk ratio (RR) was utilized to analyze the dichotomous variables and the weighted mean difference (WMD) was utilized to assess the continuous variables. Based on Der Simonian and Laird approach, the random-effects model was utilized so as to account for clinical heterogeneity due to both sampling variability and other factors such as differences in surgical skill and the numbers of procedures carried out by a surgeon. According to the overall POPF rate, the bias of potential publication was determined by carrying out informal visual inspection of funnel plots. The software of Review Manage version 5.1 (RevMan 5.1) that was downloaded from Cochrane Library was used to conduct data analysis. *P* < 0.05 was deemed as statistically important.

## Results

### Search results and baseline characteristics

A systematic review of the literature of peer-reviewed medical journals was performed to identify all studies published up to January 2017 that focused on the use of MIPD. The last search was performed on May 27th, 2017. A total of 528 potential articles published between 1994 and 2017 were initially identified from the literature searches. After the titles, abstracts and full text if necessary were reviewed, papers without according the eligibility criteria were excluded. A total of 144 studies included 37 comparative studies of MIPD and OPD, and the remaining 107 studies focused on therapeutic outcomes for MIPD (including 36 case reports and 8 video reports). The relevant literature was very scarce prior to 2004 (*n* = 3) [1, 10, 11]. After that, the articles about MIPD increased year by year. In the early years, most articles were case reports or non-control case series studies, which mainly came from Western Europe and South Asia countries. However, in the last 6 years high-volume and comparative researches were increasing gradually (Additional file 1). Growing medical centers, especially in USA, Japan and China, reported their trails in performing MIPD, from case report to case series, then to comparative studies. The highest number of publications has been from USA (*n* = 54), followed by Japan (*n* = 20), China (*n* = 19) (Additional file 2). There still had been no prospective randomized studies (RCTs) yet. Additional file 3 shows a summary of previously published original articles that without including case reports, video reports or technical reports without surgical data [12–111].

The majority of papers were single center research based on their initial experience with short-term surgical outcomes. Thirteen studies reported mid- or long-term postoperative results [15, 16, 24, 31, 55, 57, 68, 80, 86, 90, 92, 96, 107], and one study concentrated on long-term quality of life (QOL) after surgery [61]. The indications of MIPD were periampullary or pancreatic disease with one exception, which reported three cases of LPD for the treatment of locally advanced gastric cancer [19]. Four studies reported experience of MIPD involving venous resection for locally advanced disease [27, 29, 69, 97]. Five reports have inquired into the learning curve of MIPD [60, 63, 67, 70, 109]. Two studies have investigated the safety and feasibility of MIPD for elderly patients [20, 85], whereas one research have evaluated the influence of obesity on the surgical outcomes of RPD [94]. Five papers had the focus on analyzing the hospital costs of MIPD [50, 71, 84, 88, 108]. Two multi-institution researches have compared the outcomes for MIPD between low-volume hospital and high-volume center [87, 99].

**Fig. 1:**
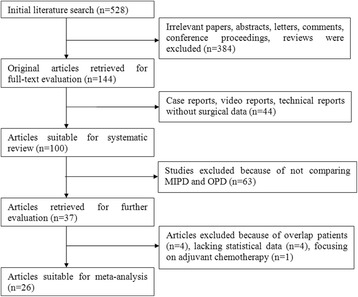
Flow chart of literature search strategies

Of these 37 cohort studies, which have compared MIPD and OPD with perioperative outcomes, four studies were excluded from the meta-analysis due to overlap patient cohorts [50, 69, 85, 107]. Another five studies based on the American National Cancer Database (NCDB) and Nationwide Inpatient Sample (NIS) were also excluded because of lacking statistical data or focusing on adjuvant chemotherapy [66, 81, 96, 104, 108]. Besides, there may be potential overlap patients with other included studies from the United States medical centers. Finally, a total of 26 comparative studies were included for quantitative meta-analysis [17, 26, 30, 31, 33–36, 47, 53, 55, 57, 61, 63–65, 68, 70, 71, 74, 83, 84, 88, 92, 106, 110]. A flow chart of the search strategies, which contains reasons of excluded studies, is illustrated in (Fig. 1).

### Characteristics and quality of studies for meta-analysis

A total of 3402 patients were included in the analysis with 1064 undergoing MIPD (31.3%) and 2338 undergoing OPD (68.7%). Total sample size for included studies ranged from 16 to 1239, with patient numbers in the MIPD arms ranging from 8 to 211. They represented an international experience (11 USA, 5 China, 2 South Korea, 2 Japan, 2 Canada, 1 UK, 1 Spain, 1 Germany and 1 France). Because MIPD was still much less performed than OPD in many centers, approximately, half of the included studies used case-match comparative analysis [31, 34, 53, 57, 61, 65, 68, 70, 83, 84, 106]. Two multi-institutional studies were found [65, 110]. Only three studies did not use the ISGPF criteria [30, 57, 61]. Nine studies used an ITT approach [26, 34–36, 53, 55, 63, 65, 68, 70, 88, 92], whereas eight were not [31, 33, 61, 64, 83, 84, 106, 110], and the remaining six were unclear [17, 30, 47, 57, 71, 74]. According to the NOS, sixteen out of the twenty-six observational studies got 6 stars, five articles got 7 stars, three articles got 8 stars and the remaining two got 9 stars. Additional file 4 presents the characteristics and quality assessment based on the NOS of the included studies. Other baseline description of these studies could be found in S3 Table 1.

**Table 1:** Results of the meta-analysis

Outcomes	No. of studies	Sample size		Heterogeneity (*P, I* ^***2***^)	Overall effect size	95% CI of overall effect	*P*
		MIPD	OPD				
Operation time (min)	12	518	1054	<0.01, 96%	WMD = 99.4	46.0 ~ 152.8	<0.01
Blood loss (mL)	9	408	845	<0.01, 83%	WMD = −0.54	−0.88 ~ −0.20	<0.01
Transfusion	15	739	1527	0.25, 18%	RR = 0.73	0.57 ~ 0.94	0.02
Time to oral intake (days)	3	172	333	0.02, 73%	WMD = −0.86	−1.90 ~ 0.18	0.11
Hospital stay (days)	11	402	829	<0.01, 76%	WMD = −3.49	−4.83 ~ −2.15	<0.01
Overall complications	24	825	1496	<0.01, 57%	RR = 0.89	0.78 ~ 1.02	0.10
POPF	25	853	1521	0.60, 0%	RR = 0.91	0.78 ~ 1.07	0.25
Severe POPF	21	983	2169	0.80, 0%	RR = 1.04	0.86 ~ 1.27	0.68
DGE	19	720	1349	0.81, 0%	RR = 0.70	0.53 ~ 0.94	0.02
PPH	10	427	839	0.46, 0%	RR = 1.18	0.79 ~ 1.78	0.42
Reoperation	14	449	817	0.49, 0%	RR = 1.02	0.70 ~ 1.49	0.92
Readmission	9	512	1219	0.46, 0%	RR = 1.16	0.96 ~ 1.40	0.13
Mortality	21	972	2158	0.99, 0%	RR = 0.81	0.51 ~ 1.30	0.39
Retrieved lymph nodes	11	421	995	<0.01, 79%	WMD = 1.13	−0.32 ~ 2.59	0.13
R0 rate	21	582	1554	0.13, 27%	RR = 1.06	1.00 ~ 1.12	0.04

### Technical details and outcomes definitions of the meta-analysis included studies

The number of surgeons who performed robot-assisted surgery was reported in 80.8% of the included studies (21/26). Most of the included studies represented the experience of single surgeon or term (Additional file 5). The majority researches included both patients with benign and malignant disease, whereas five studies only confined to malignant tumors [30, 55, 57, 61, 92]. Although MIPD has been described for all indications, even locally advanced malignant disease invading surrounding organs or portal vein system [29, 69], most studies excluded patients with adenocarcinoma involving the vascular structures or very large tumor [17, 33, 35, 36, 53, 63, 64, 68, 70, 71, 110]. The most commonly reported pancreatic anastomosis procedure were duct-to-mucosa, end-to-side pancreaticojejunostomy (PJ) with or without stents. Some studies also included cases of pancreaticogastrostomy (PG) [26, 30, 65, 106]. The surgical indications and technical details reported by the included studies are shown in Additional file 5.

### Intraoperative effects

**Fig. 2:**
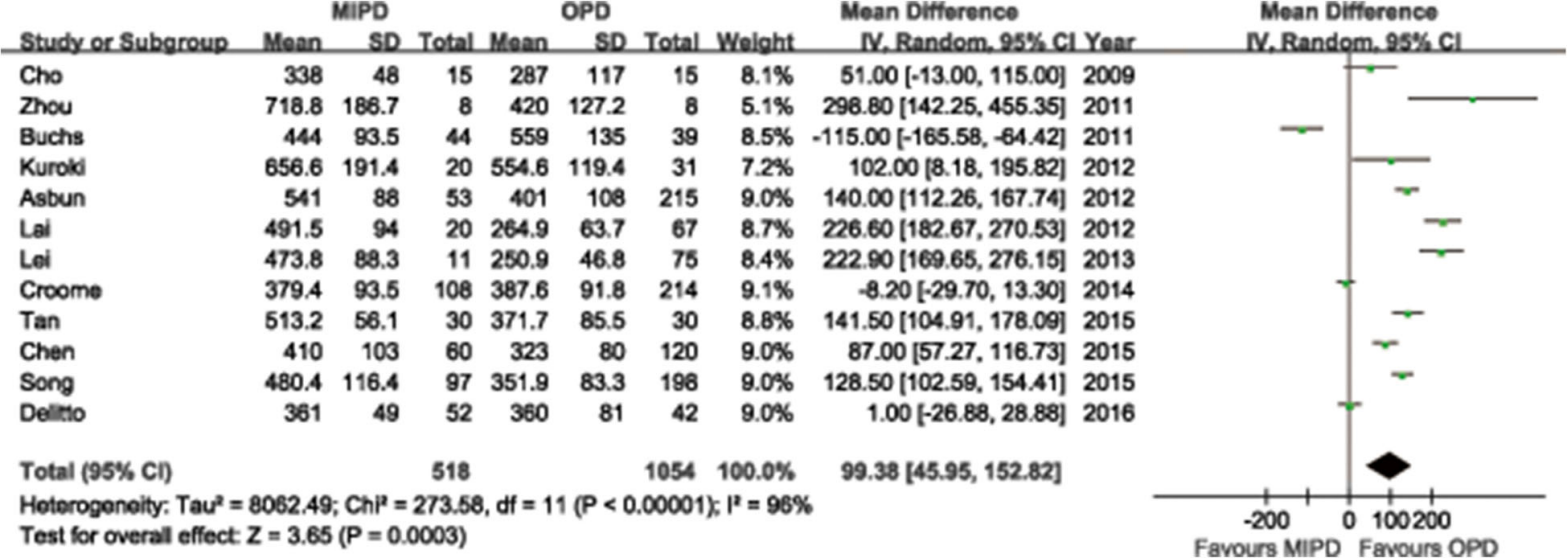
Forest plot of the meta-analysis: operation time

**Fig. 3:**
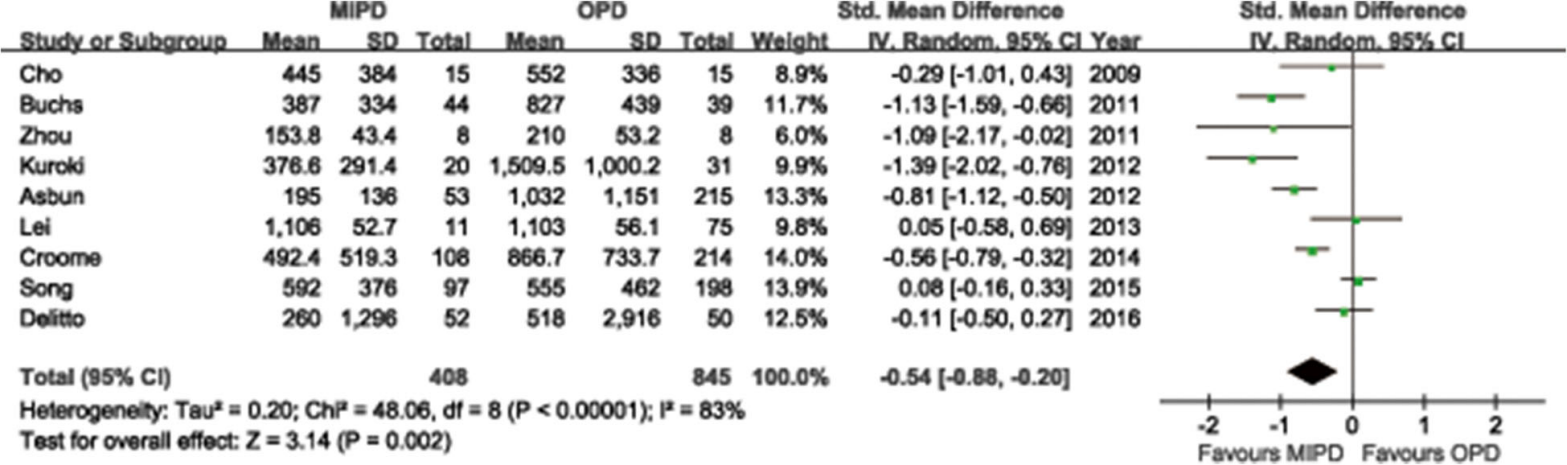
Forest plot of the meta-analysis: estimated blood loss

**Fig. 4:**
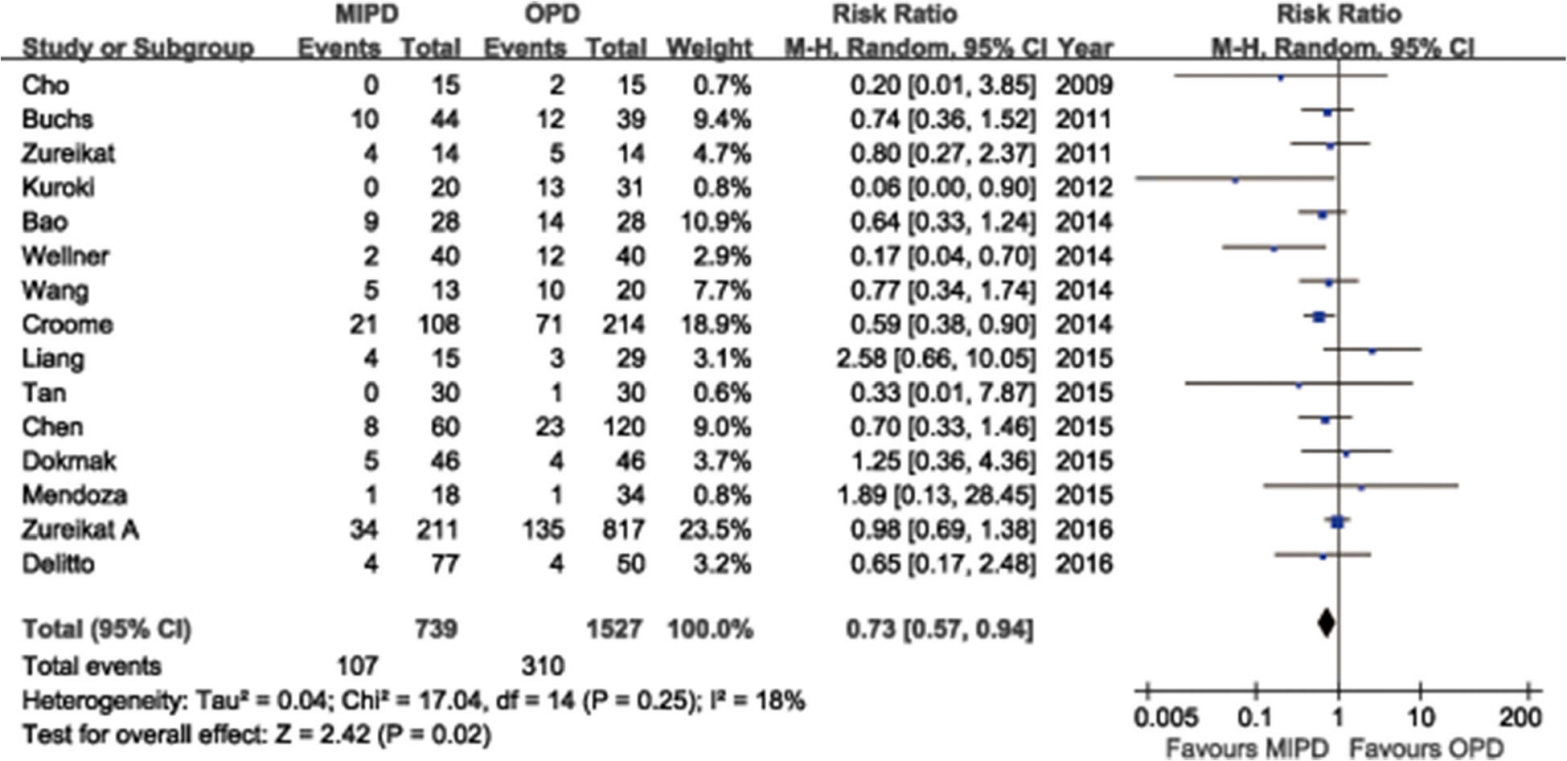
Forest plot of the meta-analysis: transfusion

Additional file 6 summarizes the reports that have evaluated the short-term surgical outcomes of MIPD, which included at least 20 cases. Conversion rates to open surgery vary substantially between 0% and 40%. Adam et al. [66] reported a conversion rate of 30% based on 983 cases of MIPD from the NCDB. The most common cause was bleeding, followed by unclear tumor margin or vein resection. Other reported causes of conversion included severe adhesions, difficult progression, and injury. We summarized the causes for conversion in the meta-analysis included studies in Additional file 7. The mean or median OP ranged from 276 to 657 min observed in all systematic review studies. The majority of the researches considered applicable for the meta-analysis revealed a longer OP for MIPD than for OPD. Twelve studies reported OP with mean and SDs, the pooled-data showed a statistically significant longer OP for MIPD (WMD = 99.4 min; 95%CI: 46.0 ~ 152.8, *P* < 0.01) (Fig. 2). Another thirteen studies reported OP with median and range, seven of which revealed a significant long OP in MIPD compared to OPD in their individual research. Two studies reported shorter OP in MIPD, but in these series, OP for OPD appeared to be longer than that usually reported [26, 65] (Additional file 8). The mean or median intraoperative EBL ranged from 65 to 842 ml and the recorded numerical data ranged from 30 to 8500 mL in all reviewed studies (Additional file 6). The meta-analysis showed the EBL was significantly lower in the MIPD compared with the OPD group (WMD = −0.54 ml; 95% CI, −0.88 ~ −0.20 ml; *P* < 0.01) (Fig. 3). Another thirteen studies reported EBL with median and range, the majority of which revealed a significant less EBL in MIPD compared to OPD in their individual research (Additional file 8). Transfusion rates were reported in two reviewed studies with a range from 0% to 32%. And the quantitative meta-analysis revealed that MIPD group have a lower transfusion rate than OPD group (RR = 0.73, 95%CI: 0.57 ~ 0.94, *P* = 0.02) (Fig. 4). The intraoperative effect outcomes of the quantitative meta-analysis are summarized in Table 1.

### Postoperative recovery

**Fig. 5:**
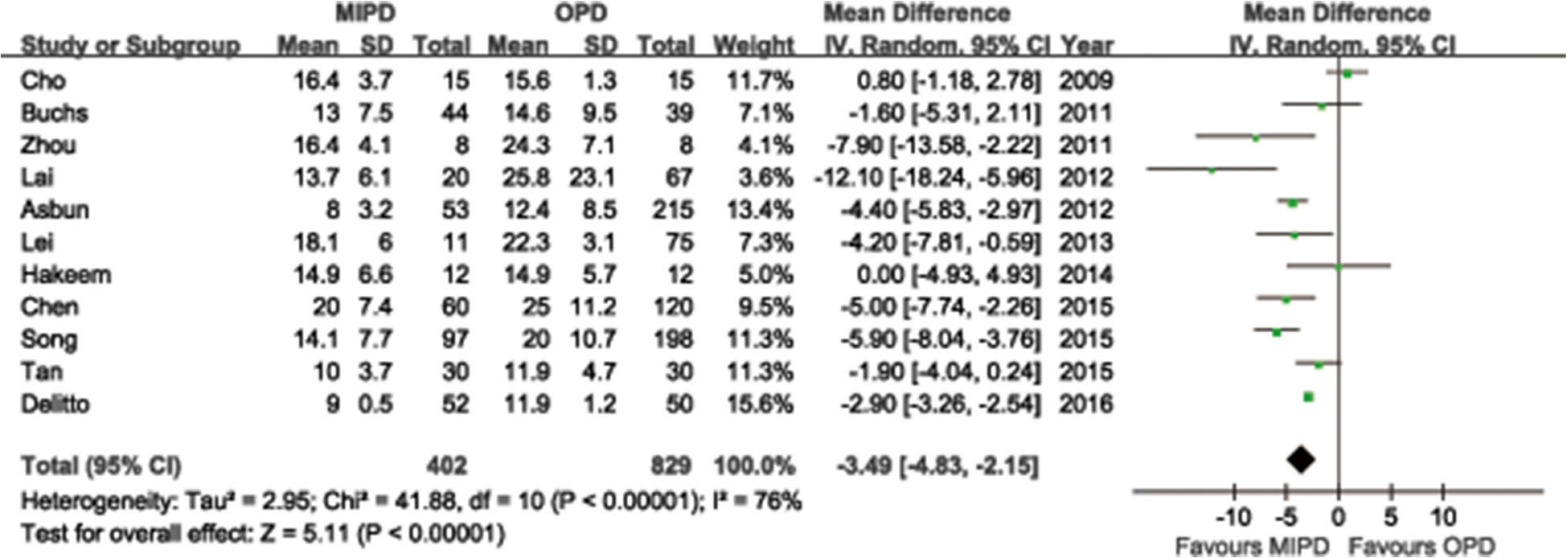
Forest plot of the meta-analysis: length of hospital stay

Only two studies reported the length of ICU stay, and the pooled-data showed a shorter length of ICU stay after MIPD than OPD (WMD = −2.08 days; 95% CI: -2.88 ~ −1.29, *P* < 0.01). Two studies reported the times of analgesic injection to evaluate the postoperative pain, and the pooled-data showed less analgesic use of MIPD than OPD (WMD = −3.72; 95% CI: -6.05 ~ −1.40, *P* < 0.01). Besides, Wang et al. [64] reported less total 7-day analgesic usage in MIPD, but the difference was not significant (*P* = 0.08). Tan et al. [84] reported less visual analogue score (VAS) on postoperative day 4 in the LPD group (*P* = 0.01). Other two studies only revealed analgesic usage tended to favor the MIPD group but was not statistically significant [53, 71]. Zhou et al. [30] reported early time to begin walk (*P* < 0.01) and Chen et al. [68] reported early time to off-bed activities (P < 0.01) and bowel movement (P < 0.01) after RPD, whereas Tan et al. [84] reported early time to return of bowel sounds (*P* < 0.01) after LPD. As for quantitative meta-analysis, only three studies with data available reported the postoperative time to restart oral intake. Analysis of the pooled data revealed early time to oral intake in MIPD group, but the difference did not reach statistically significant (WMD = −0.86 days; 95%CI: -1.90 ~ 0.18, *P* = 0.11). Forty-nine of systematic reviewed studies reported on mean or median length of hospital stay, which ranged from 6 to 25 days, respectively, and the recorded length ranged from 3 to 118 days (Additional file 6). Eleven studies with mean and SDs considered suitable for the meta-analysis which indicated a shorter hospital stay in the MIPD group than OPD group (WMD = −3.49 days; 95%CI: -4.83 ~ −2.15, P < 0.01) (Fig. 5). All postoperative outcomes the quantitative meta-analysis are also summarized in Table 1.

### Morbidity and mortality

**Fig. 6:**
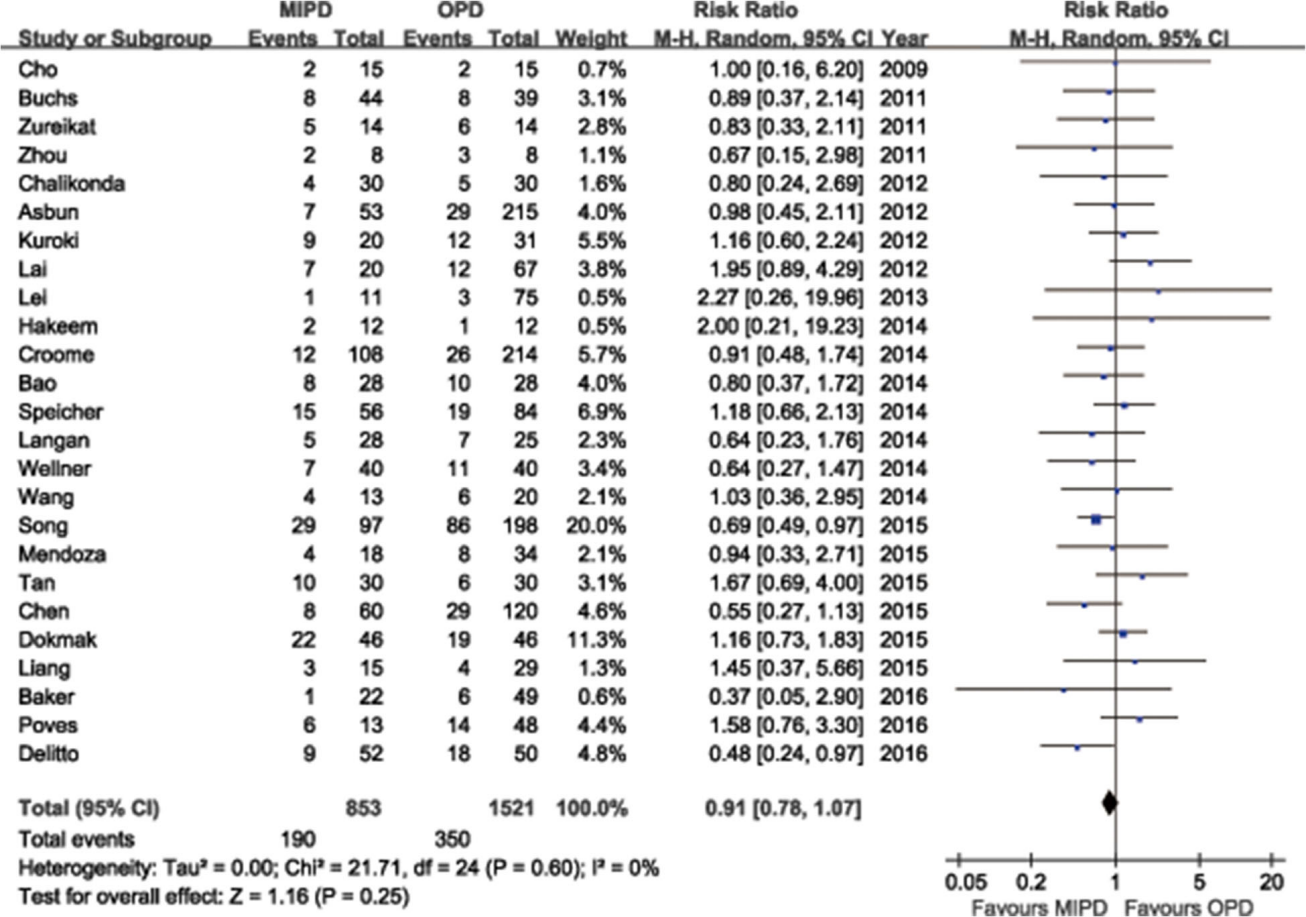
Forest plot of the meta-analysis: overall POPF

The majority of the included studies reported on adverse events with a range of 18.2%-87.5% for MIPD and 8.0%–92.9% for OPD. According to the results of the meta-analysis, though the incidence of postoperative complications tended to favor the MIPD group, but there was not statistically significant difference (RR = 0.89, 95%CI: 0.78 ~ 1.02, *P* = 0.10). We summarized the specific postoperative surgical and medical complications based on all reviewed researched in Table 2 and Table 3. The most common surgical complication was POPF, followed by DGE, PPH, fluid collection or abscess, wound infection, bile leakage, ileus, et al. And the most common medical complication was respiratory complications, especially pulmonary infection, followed by cardiovascular complications and renal complications. The total POPF rates vary substantially between 3.8% and 50% observed in all systematic reviewed studies (Additional file 6). As to quantitative meta-analysis, no significant difference was found in pooled data of the meta-analysis between the MIPD group and OPD group in regard to the rate of overall POPF (RR = 0.91, 95%CI: 0.78 ~ 1.07, *P* = 0.25) (Fig. 6), as was the severe POPF rates (RR = 1.04, 95%CI: 0.86 ~ 1.27, *P* = 0.68), which was defined as grade B and above according to the ISGPF. The meta-analysis also did not find any statistically significant differences between groups regarding other complications, such as DGE and PPH. Fourteen studies reported reoperation cases, and there was no significant difference in reoperation rate (RR = 1.02, 95%CI: 0.70 ~ 1.49, *P* = 0.92). The specific reoperation reasons observed in the meta-analysis included studies are summarized in Additional file 9. Nine studies reported readmission cases, and there was also no significant difference in readmission rate (RR = 1.16, 95%CI: 0.96 ~ 1.40, *P* = 0.13). The overall perioperative mortality rates range from 0%–6.9% in all systematic reviewed studies (Additional file 6). As for quantitative analysis of perioperative mortality, the meta-analysis of cohorts demonstrated no significant difference between the two groups (RR = 0.81, 95%CI: 0.51 ~ 1.30, *P* = 0.39). The specific death reasons in the meta-analysis studies are summarized in Additional file 10. The grade C POPFs were primary causes of postoperative reoperation and death. The morbidity and mortality outcomes of the quantitative meta-analysis are also summarized in Table 1.

**Table 2:** Surgical complications of MIPD in reviewed studied (list by constituent ratio)

Complications	Total events (*n* = 1003)
POPF	372 (37.1%)
DGE	255 (25.4%)
PPH	141 (14.1%)
Fluid collection/abscess	84 (8.4%)
Wound complications	60 (6.0%)
Bile leakage	56 (5.6%)
Ileus	17 (1.7%)
Chyle leakage	5 (0.5%)
Gastrointestinal anastomotic leakage	4 (0.4%)
Peptic ulcer	3 (0.3%)
Other	6 (0.6%)

Other complications included colitis (*n* = 1), colon perforation (*n* = 1), bowel ischemia (*n* = 1), PV thrombus (*n* = 1), dumping syndrome (*n* = 1), trocar site bleeding (*n* = 1)

**Table 3:** Medical complications of MIPD in reviewed studied (list by constituent ratio)

Complications	Total events (*n* = 199)
Respiratory	98 (49.2%)
Cardiovascular	69 (34.7%)
Renal	18 (9.0%)
Deep venous thrombosis	10 (5.0%)
Other	4 (2.0%)

Respiratory: included pulmonary infection, pneumonias, pleural effusion, pulmonary embolism, pulmonary edema, atelectasis, lung failure, ARDS; Cardiovascular: included heart failure, myocardial infarction, arrhythmia, hypotension, acute coronary syndrome; Renal: included renal failure, urinary infection, urinary retention, renal insufficiency; other complications included thrombocytopenia (*n* = 1), anemia (*n* = 1), hepatic insufficiency (*n* = 2)

### Oncologic outcomes and long-term survival

**Fig. 7:**
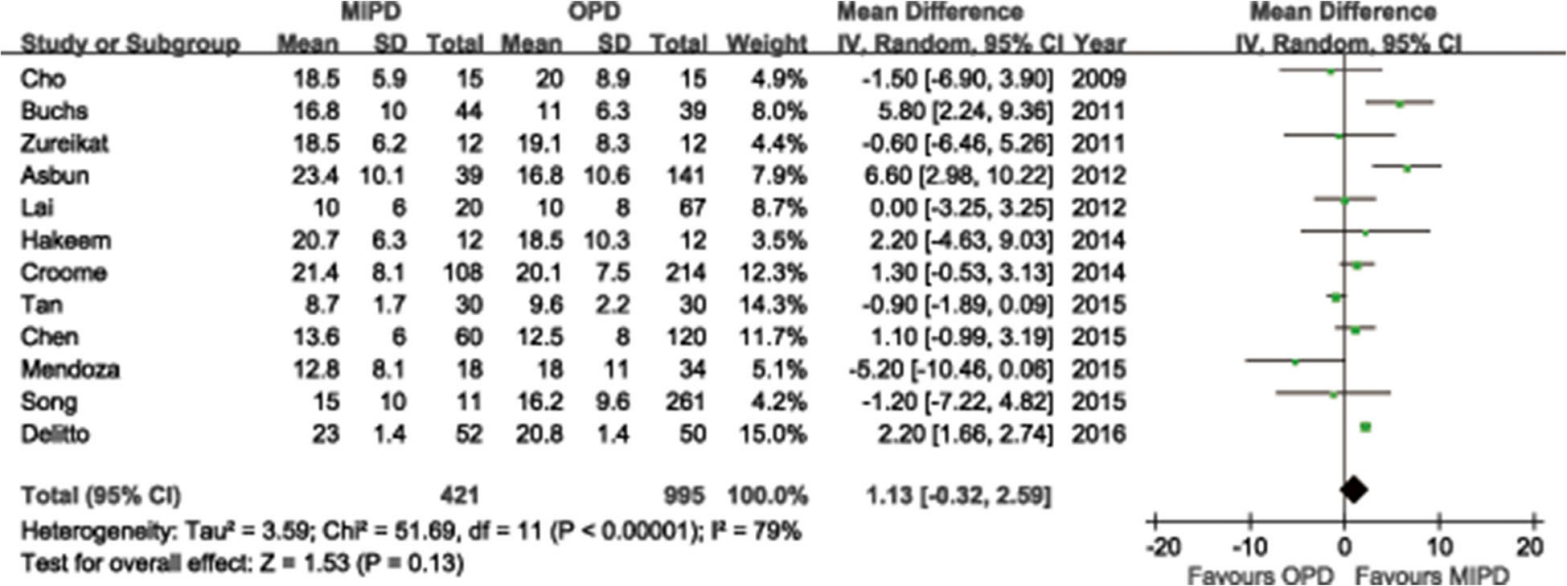
Forest plot of the meta-analysis: retrieved lymph nodes

The mean or median number of retrieved lymph nodes ranged from 6 to 32 observed in systematic review studies (Additional file 6). For meta-analysis, all studies reported tumor histology in both MIPD and OPD groups. The difference in the mean number of retrieved lymph nodes was not significant in the pooled data with a tendency of more in MIPD group than in OPD group (WMD = 1.13; 95%CI: -0.32 ~ 2.59, P = 0.13) (Fig. 7). As for margin status, only malignant cases were considered, thus twenty-one studies reported margin status. On pooling the results, the negative margins (R0) rate was higher in MIPD group than that in OPD group (RR = 1.06, 95%CI: 1.00 ~ 1.12, *P* = 0.04). Survival status is the most critical outcome for evaluating surgical interventions in oncological therapy. However, limited long-term survival outcomes are available from this review. We summarized the information available about long-term survival outcomes in Table 4. Only three studies reported the cancer recurrence, and the difference between MIPD group and OPD group was not significant (RR = 0.78, 95%CI: 0.61 ~ 1.01, *P* = 0.06). Five studies reported comparable survival time or rate between two groups [55, 57, 68, 83, 92]. However, the meta-analysis of survival rate cannot be done due to limited data.

**Table 4:** Summary of Recurrence and Long-term Survivals

Author	Group	Malignant case	Follow-up (month)	Recurrence	Survival (time: month; rate: %)
Zureikat [31]	MIPD	12	9.5(4–21)	1	NR
Croome [55]	MIPD	108	16.5	44	MOS: 25.3 m
	OPD	214	15.1	113	MOS: 21.8 m
Hakeem [57]	MIPD	12	46.8(13.0–73.7)	2	1, 3, 5y–DFS: 100; 92, 83, 5y–OS: 100, 83, 83
	OPD	12	56.0(1.0–97.4)	2	1, 3, 5y–DFS: 100; 92, 75, 5y–OS: 75, 58, 50
Song [83]	MIPD	11	NR	NR	5y–OS: 53.6
	OPD	222	NR	NR	5y–OS: 28.8
Chen [68]	MIPD	19	22 ± 10	NR	MDFS: 14.0 m; MOS: 23.0 m
	OPD	39	21 ± 8	NR	MDFS: 13.0 m; MOS: 22.0 m
Delitto [92]	MIPD	52	NR	NR	MOS: 27.9 m; MOS: 20.7 m*
	OPD	50	NR	NR	MOS: 23.5 m; MOS: 21.1 m*
Palanivelu [15]	MIPD	49	36.5	NR	MOS: 49 m; 5y–OS: 30.4
Pugliese [16]	MIPD	11	32(12–45)	7	MDFS: 11 m; MOS: 18 m
Senthilnathan [80]	MIPD	130	24	NR	MOS: 33 m; 5y–OS:29.4
Wang [86]	MIPD	16	18(8–48)	10	MDFS: m16; MOS: 19 m
Coratti [90]	MIPD	41	15.8(2–47)	12	MOS: 40 m; 1, 2, 3y–OS: 81, 69, 55
Kantor [96]	MIPD	828	18	NR	MOS: 20.7 m
	OPD	7385		NR	MOS: 20.9 m
Stauffer [107]	MIPD	58	19.6 ± 17.4	NR	MOS: 18.5 m; 1, 2, 3, 4, 5y–OS: 66.5, 43.3, 43.3, 38.5, 32.1
	OPD	193	24.5 ± 27.4	NR	MOS: 20.3 m; 1, 2, 3, 4, 5y–OS: 67.5, 40.2, 24.3, 17.1, 15.3

Follow-up time were shown as median (range) or median only; DFS: disease-free survival rate; OS: overall survival rate; MDFS: median disease-free survival time; MOS: median overall survival time; y: year; *special for PDAC; NR: not report

### Publication bias

**Fig. 8:**
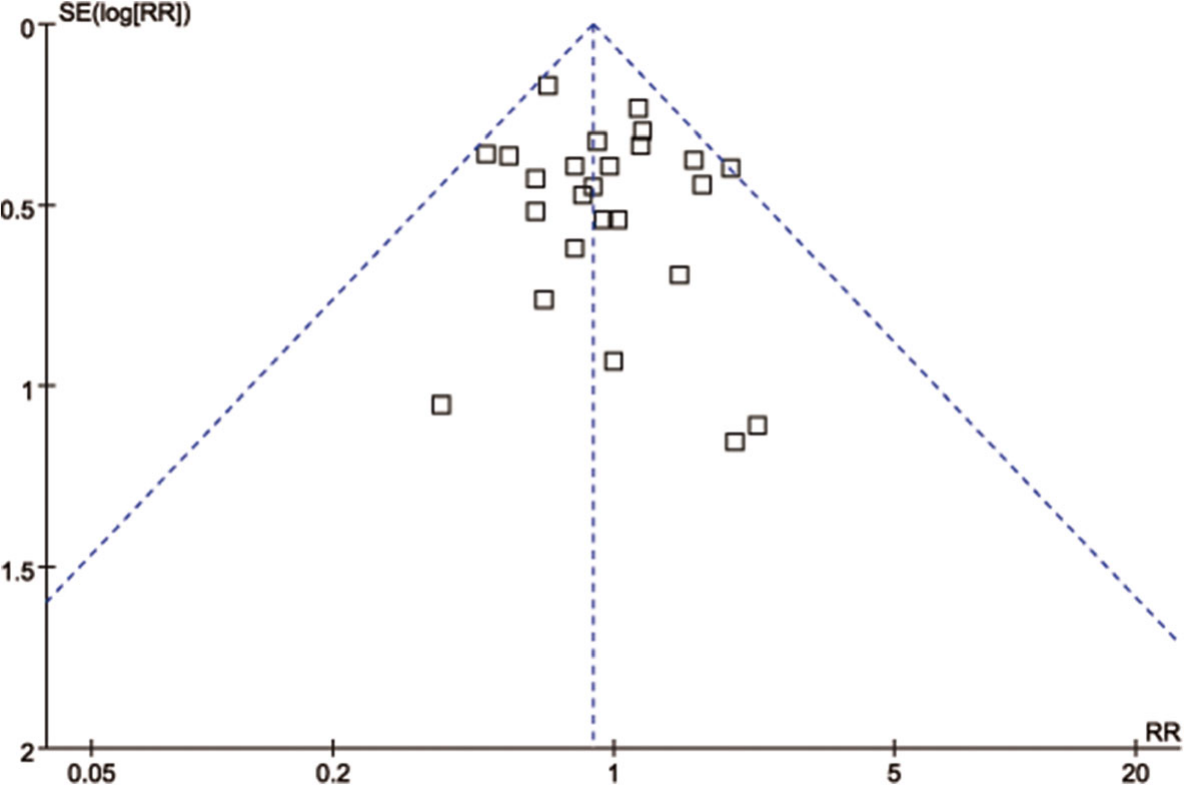
Funnel plot of the overall POPF rates

As showed in Fig. 8, the funnel plot for studies reporting the RRs of POPF was used to detect the publication bias. All the plots standing for the studies fall within the 95% CI axis and distributed symmetrically. This result suggested the publication bias was minimal and acceptable.

## Discussion

It is well-known that PD is one of the most complicated abdominal surgeries, and the completion of dissection of the porta hepatis, extensive lymphadenectomy and uncinate process detachment intracorporeally is technically high-demanding. This inevitably leads to the traditional view that minimally invasive surgical approaches are relatively contraindicated in the setting of PD. With the accumulation of minimally invasive surgical experience, more surgeons introduced MIPD and some of them even adapted it as a conventional procedure. Our systematic review also revealed a trend of steeply increasing surgeons performing MIPD around the world. By reviewing some overlapped studies from the same centers, we found surgeons were improving their surgical techniques in MIPD, from benign disease to malignances, from patients with good condition to those with bad condition. Speicher et al. reported their train projects for surgeons in adaption of LPD [63]. These all suggested MIPD was not a trick specially played by the talents but can be adapted by more surgeons with fully training and practices.

The conversion rate to OPD appears to be higher when comparing to other minimally invasive gastroenterological surgery. Reasons attributed to conversion including larger tumors, pancreatic adenocarcinoma or chronic pancreatitis, adhesions to vascular structures, uncontrollable bleeding et al. [65]. The reported rates based this comprehensive review for conversion to open approach range from 0% to 40%. Such substantial difference in conversion rates reported was mainly attributed to the learning curve of the surgeon in obtaining proficiency and the discrepancy in patient selection. Furthermore, the liberalization of intraoperative decision to convert varies from individual to individual. Wellner et al. argued that the minimally invasive approach should not be pursued at the cost of major increase in technical difficulties or operation time [65], leading their decision to conversion more liberalized. Some excellent surgeons argued that MIPD could performed for all indications, even locally advanced malignant disease invading surrounding organs or portal vein system [27, 29, 69, 97]. However, they also admitted that major venous resection intracorporeally was only feasible in selected patients and advocated that extensive experience with complex laparoscopic pancreatic resection and reconstruction should prior to attempt such procedure.

In our pooled analysis, the mean OP during MIPD was significantly longer than that during OPD. In spite of the considerable heterogeneity, the prolonged OP in MIPD was shown in the majority researches included. In addition to the complicated resection phase, the lengthy gastroenterological, pancreatic and biliary anastomoses are especially technically demanding and time-consuming. Given the fact that the majority studies included in the meta-analysis reported on their initial experience, OP could be expected to decrease or even close to OPD when more experience is gained. For these approaches it will take a surgeon several years to overcome the learning curve and achieve high- quality outcomes [67].

A hybrid or laparoscopic assisted approach, in which the anastomoses are performed via a small laparotomy, might facilitate MIPD and be useful to accumulate experience [64, 65]. Some publications advised that minimally invasive procedures can be safely performed when using a hybrid method before starting with totally intracorporeal surgery. Performing MIPD using hybrid method in initial series helped to safely shift to totally intracorporeal method [31, 63]. Nevertheless, intracorporeally anastomosis, especially for PJ, should base on progressive practice. Practice on the simulator then to animal models for simple suture under laparoscopy, and finally to intracorporeal gastrointestinal anastomosis can effectively shorten the learning curve. Some researcher did the resection laparoscopically, then introduced robot to promote the reconstruction, which was so-called robotic and laparoscopic hybrid PD, demonstrating favorite outcomes [24, 59].

Intraoperative EBL is an important index to judge the safety in operation, which was shown in the pooled data to be lower in MIPD, so was the need for transfusions. The need for transfusion arises due to excessive blood loss and the two outcomes are therefore correlated. The EBL is subjective and tended to bias, whereas transfusion is an objective outcome. However, the definition of blood loss and criteria for blood transfusion were not explicitly reported in any of the included studies. Besides, there were serious skewedness in EBL between studies (recorded numerical data ranged from 50 to 1800 mL in review studies), with significant heterogeneity mainly due to different methods of EBL. Therefore, the results should be interpreted prudently and need further study to confirm.

The substantial risk of perioperative mortality and morbidity remains a major concern to limit the development of MIPD. Although surgical safety had been improved gradually mainly related to centralization, the conventional OPD was still associated with postoperative morbidity rates of 23% to 66% and mortality rates of 3% to 5% even in high-volume centers [112–114]. The range of overall morbidity and mortality rate of MIPD observed from the series analyzed were 18.2% to 87.5% and 0% to 6.9%, respectively, which is comparable to the OPD literature. And the quantitative meta-analysis also demonstrated no significant differences with regard to perioperative mortality and morbidity between two groups. POPFs remain one of the most threatening complications after PD, with rates ranging from 4% to 33% in series from expert centers [115]. The reviewed series in this study also revealed a comparable POPFs range of 3.8% to 50%. Our quantitative meta-analysis demonstrated the comparable complication rates of POPF, especially the severe POPF, DGE and PPH in the MIPD versus the OPD group, and the low heterogeneity encourages us to believe that MIPD could be as safe as OPD. PD involved in multiple systems and would cause more medical complications than other operations. It was observed from the reviewed studies that respiratory complications were the most commonly medical complications, mainly pulmonary infection, followed by cardiovascular ones. Less pain and faster recovery obtained from minimally invasive approach bring about mild inflammatory reaction, which may contribute to the maintaining of normal pulmonary function [116]. It was also reported that more intraoperative blood loss and transfusion could increase respiratory complication risks [117]. In our study, MIPD had significantly less blood loss and transfusion, which helped to reduce respiratory complications. Therefore, MIPD may be more suitable to patients with severe impaired pulmonary function [118]. It was conceivable that mortality was similar between groups due to the similar complication and reoperation rates. Summary for causes of reoperation and death based on the reviewed studies revealed that the common causes were abdominal hemorrhage, abscess, and sepsis mainly due to grade C POPF. These outcomes turned out to be equivalent between MIPD and OPD in our meta-analysis. Likewise, Gagner et al. argued that a pancreatic fistula from a failed pancreatic anastomosis can portend serious complications, including bleeding, sepsis, and death [119]. The two-layer, end-to-side, duct-to-mucosa PJ was the most commonly used pancreatic anastomosis approach. In order to reduce PF, various modified techniques have been reported, such as so-called “pancreas-hanging maneuver” [23], linear stapler pancreatic stump closure [37], “double jejunal loop reconstruction” [48], “barbed suture PJ” [56], “dunking PJ using mattress sutures” [54], “intussuscepting PJ” [58], “two transpancreatic sutures with buttresses” [98], “purse-string suture without pancreatic parenchymal stitches” [95], “laparoscopic-adapted Blumgart PJ” [106], “imbedding PJ” [111], et al. or even intracorporeal PG [73]. Although the authors reported favorable results, the exact value of these techniques remains unclear and needs further studies to confirm.

As for postoperative recovery, the pooled data demonstrates mixed outcomes with less time spent in ICU and less usage of analgesic in MIPD group than OPD group, while only moderate earlier time to oral intake in the MIPD group without significant difference, indicating MIPD likely enhanced the recovery of intestinal function. Reduced use of analgesic drugs, shortened time of abdominal cavity exposure, and earlier postoperative activities are considered to be the main reasons for earlier gastrointestinal recovery from MIPD.

As the removal of a sufficient amount of lymph nodes can enhance the accuracy of staging and regional disease control, appropriate lymphadenectomy is one of the most crucial steps in radical pancreatic surgery. The mean or median number of retrieved lymph nodes ranged from 6 to 32 observed in systematic review studies and the quantitative meta-analysis showed no significant difference between MIPD and OPD groups. It is well-documented that only about 20% eligibility rate for surgery among patients with pancreatic cancer when diagnosed. It has also been suggested that even patients who are eligible for surgery preoperatively cannot be offered potentially curative resection mainly due to vascular invasion [120, 121]. Because few reports have compared long-term survival outcomes with other methods, a meta-analysis of long-term oncologic results comparing MIPD versus OPD could not be done due to limited data. Because of most surgeons performed their initial minimally invasive Whipple procedures in patients with resectable small ampullary, distal bile duct or duodenal adenocarcinoma, and the prognosis of periampullary tumors with different pathological types is very different. Therefore, the oncological efficiency of MIPD should ideally be assessed in a randomized controlled trial.

Our researches have some limitations as below: (1) No RCTs were included: All the evidence was derived from observational studies with either concurrent or historical controls, and the use of some historical control groups that may be inadequately matched to the intervention group and because of poor or selective outcome reporting. (2) Selection bias: Most hospitals are still in the learning curve now and during that phase only the most ideal patients are selected. (3) Clinical heterogeneity: The variety of postoperative management, discharge criteria and application of fast track protocol et al. will lead to clinical heterogeneity and a sequence of biases. (4) Lacking long-term outcomes: large studies on the long-term outcomes, such as oncological efficiency, especially for long-term survival and quality of life (QOL) are scarce.

## Conclusions

Our systematic review and meta-analysis demonstrates that MIPD is technically feasible and safety, especially in experienced hands. MIPD alleviates surgical traumas and enhanced postoperative recovery. Account for the inherent limitations of our study, our findings should also be interpreted with cautions. Robust prospective comparative studies and randomized clinical trials are awaited to bring more high quality evidences.

## Additional files

Additional file 1: Number of original publications concerning MIPD by year according to the study design (abstracts, letters, comments, reviews were not included). (TIFF 56 kb)

Additional file 2: Number of total publications concerning MIPD by year according to the place of origin (the majority North American studies came from USA; other regions included Turkey and Brazil). (TIFF 67 kb)

Additional file 3: Published articles of MIPD. (DOCX 52 kb)

Additional file 4: Baseline and quality assessment based on the NOS for meta-analysis included studies. (DOCX 30 kb)

Additional file 5: Summary of indications and laparoscopic technical details in the meta-analysis included studies. (DOCX 26 kb)

Additional file 6: Summary of perioperative outcomes in reviewed studies with more than twenty cases of MIPD. (DOCX 37 kb)

Additional file 7: Summary of reasons for conversion. (DOCX 22 kb)

Additional file 8: Summary of perioperative outcomes in studies without quantitative data presenting mean and standard deviation. (DOCX 24 kb)

Additional file 9: Summary of the specific reoperation reasons. (DOCX 22 kb)

Additional file 10: Summary of the specific perioperative death reasons. (DOCX 23 kb)

## Abbreviations

CI: Confidence interval
DGE: Delayed gastric emptying
EBL: Estimated blood loss
ISGPF: International Study Group for Pancreatic Fistula
ITT: Intention-to-treat
LOS: Length of hospital stay
LPD: Laparoscopic pancreatoduodenectomy
MIPD: Minimally invasive pancreatoduodenectomy
NOS: Newcastle-Ottawa Quality Assessment Scale
OP: Operation time
OPD: Open pancreatoduodenectomy
PD: Pancreatoduodenectomy
PG: Pancreaticogastrostomy
PJ: Pancreaticojejunostomy
POPF: Postoperative pancreatic fistula
PPH: Post-pancreatectomy hemorrhage
QOL: Quality of life
RCT: Prospective randomized studies
RLN: Retrieved lymph nodes
RPD: Robotic pancreatoduodenectomy
RR: Risk ratio
SD: Standard deviation
WMD: Weighted mean difference

## References

[1] Gagner M, Pomp A (1994). Laparoscopic pylorus-preserving pancreatoduodenectomy. Surg Endosc.

[2] de Rooij T, Klompmaker S, Abu Hilal M, Kendrick ML, Busch OR, Besselink MG (2016). Laparoscopic pancreatic surgery for benign and malignant disease. Nature reviews Gastroenterology & hepatology.

[3] Gagner M, Pomp A (1997). Laparoscopic pancreatic resection: is it worthwhile?. Journal of gastrointestinal surgery : official journal of the Society for Surgery of the Alimentary Tract.

[4] Kendrick ML (2012). Laparoscopic and robotic resection for pancreatic cancer. Cancer journal (Sudbury, Mass).

[5] Hozo SP, Djulbegovic B, Hozo I (2005). Estimating the mean and variance from the median, range, and the size of a sample. BMC Med Res Methodol.

[6] Grobmyer SR, Pieracci FM, Allen PJ, Brennan MF, Jaques DP (2007). Defining morbidity after pancreaticoduodenectomy: use of a prospective complication grading system. J Am Coll Surg.

[7] Bassi C, Dervenis C, Butturini G, Fingerhut A, Yeo C, Izbicki J, Neoptolemos J, Sarr M, Traverso W, Buchler M (2005). Postoperative pancreatic fistula: an international study group (ISGPF) definition. Surgery.

[8] Wente MN, Bassi C, Dervenis C, Fingerhut A, Gouma DJ, Izbicki JR, Neoptolemos JP, Padbury RT, Sarr MG, Traverso LW (2007). Delayed gastric emptying (DGE) after pancreatic surgery: a suggested definition by the international study Group of Pancreatic Surgery (ISGPS). Surgery.

[9] Wente MN, Veit JA, Bassi C, Dervenis C, Fingerhut A, Gouma DJ, Izbicki JR, Neoptolemos JP, Padbury RT, Sarr MG (2007). Postpancreatectomy hemorrhage (PPH): an international study Group of Pancreatic Surgery (ISGPS) definition. Surgery.

[10] Uyama I, Ogiwara H, Iida S, Takahara T, Furuta T, Kikuchi K (1996). Laparoscopic minilaparotomy pancreaticoduodenectomy with lymphadenectomy using an abdominal wall-lift method. Surgical laparoscopy & endoscopy.

[11] Gagner M, Pomp A, Herrera MF (1996). Early experience with laparoscopic resections of islet cell tumors. Surgery.

[12] Dulucq JL, Wintringer P, Stabilini C, Feryn T, Perissat J, Mahajna A (2005). Are major laparoscopic pancreatic resections worthwhile? A prospective study of 32 patients in a single institution. Surg Endosc.

[13] Staudacher C, Orsenigo E, Baccari P, Di Palo S, Crippa S (2005). Laparoscopic assisted duodenopancreatectomy. Surg Endosc.

[14] Dulucq JL, Wintringer P, Mahajna A (2006). Laparoscopic pancreaticoduodenectomy for benign and malignant diseases. Surg Endosc.

[15] Palanivelu C, Jani K, Senthilnathan P, Parthasarathi R, Rajapandian S, Madhankumar MV (2007). Laparoscopic pancreaticoduodenectomy: technique and outcomes. J Am Coll Surg.

[16] Pugliese R, Scandroglio I, Sansonna F, Maggioni D, Costanzi A, Citterio D, Ferrari GC, Di Lernia S, Magistro C: Laparoscopic pancreaticoduodenectomy: a retrospective review of 19 cases. Surgical laparoscopy, endoscopy & percutaneous techniques 2008, 18(1):13–18. 10.1097/SLE.0b013e3181581609 18287976

[17] Cho A, Yamamoto H, Nagata M, Takiguchi N, Shimada H, Kainuma O, Souda H, Gunji H, Miyazaki A, Ikeda A (2009). Comparison of laparoscopy-assisted and open pylorus-preserving pancreaticoduodenectomy for periampullary disease. Am J Surg.

[18] Palanivelu C, Rajan PS, Rangarajan M, Vaithiswaran V, Senthilnathan P, Parthasarathi R, Praveen Raj P (2009). Evolution in techniques of laparoscopic pancreaticoduodenectomy: a decade long experience from a tertiary center. J Hepato-Biliary-Pancreat Surg.

[19] Shinohara T, Uyama I, Kanaya S, Inaba K, Isogaki J, Horiguchi A, Miyakawa S (2009). Totally laparoscopic pancreaticoduodenectomy for locally advanced gastric cancer. Langenbeck's Arch Surg.

[20] Buchs NC, Addeo P, Bianco FM, Gangemi A, Ayloo SM, Giulianotti PC (2010). Outcomes of robot-assisted pancreaticoduodenectomy in patients older than 70 years: a comparative study. World J Surg.

[21] Giulianotti PC, Sbrana F, Bianco FM, Elli EF, Shah G, Addeo P, Caravaglios G, Coratti A (2010). Robot-assisted laparoscopic pancreatic surgery: single-surgeon experience. Surg Endosc.

[22] Kendrick ML, Cusati D (2010). Total laparoscopic pancreaticoduodenectomy: feasibility and outcome in an early experience. Archives of surgery (Chicago, Ill : 1960).

[23] Kuroki T, Tajima Y, Kitasato A, Adachi T, Kanematsu T (2010). Pancreas-hanging maneuver in laparoscopic pancreaticoduodenectomy: a new technique for the safe resection of the pancreas head. Surg Endosc.

[24] Narula VK, Mikami DJ, Melvin WS (2010). Robotic and laparoscopic pancreaticoduodenectomy: a hybrid approach. Pancreas.

[25] Ammori BJ, Ayiomamitis GD (2011). Laparoscopic pancreaticoduodenectomy and distal pancreatectomy: a UK experience and a systematic review of the literature. Surg Endosc.

[26] Buchs NC, Addeo P, Bianco FM, Ayloo S, Benedetti E, Giulianotti PC (2011). Robotic versus open pancreaticoduodenectomy: a comparative study at a single institution. World J Surg.

[27] Giulianotti PC, Addeo P, Buchs NC, Ayloo SM, Bianco FM (2011). Robotic extended pancreatectomy with vascular resection for locally advanced pancreatic tumors. Pancreas.

[28] Horiguchi A, Uyama I, Miyakawa S (2011). Robot-assisted laparoscopic pancreaticoduodenectomy. Journal of hepato-biliary-pancreatic sciences.

[29] Kendrick ML, Sclabas GM (2011). Major venous resection during total laparoscopic pancreaticoduodenectomy. HPB : the official journal of the International Hepato Pancreato Biliary Association.

[30] Zhou NX, Chen JZ, Liu Q, Zhang X, Wang Z, Ren S, Chen XF (2011). Outcomes of pancreatoduodenectomy with robotic surgery versus open surgery. The international journal of medical robotics + computer assisted surgery : MRCAS.

[31] Zureikat AH, Breaux JA, Steel JL, Hughes SJ (2011). Can laparoscopic pancreaticoduodenectomy be safely implemented?. Journal of gastrointestinal surgery : official journal of the Society for Surgery of the Alimentary Tract.

[32] Zureikat AH, Nguyen KT, Bartlett DL, Zeh HJ, Moser AJ (2011). Robotic-assisted major pancreatic resection and reconstruction. Archives of surgery (Chicago, Ill : 1960).

[33] Asbun HJ, Stauffer JA (2012). Laparoscopic vs open pancreaticoduodenectomy: overall outcomes and severity of complications using the accordion severity grading system. J Am Coll Surg.

[34] Chalikonda S, Aguilar-Saavedra JR, Walsh RM (2012). Laparoscopic robotic-assisted pancreaticoduodenectomy: a case-matched comparison with open resection. Surg Endosc.

[35] Kuroki T, Adachi T, Okamoto T, Kanematsu T (2012). A non-randomized comparative study of laparoscopy-assisted pancreaticoduodenectomy and open pancreaticoduodenectomy. Hepato-Gastroenterology.

[36] Lai EC, Yang GP, Tang CN (2012). Robot-assisted laparoscopic pancreaticoduodenectomy versus open pancreaticoduodenectomy--a comparative study. International journal of surgery (London, England).

[37] Nakamura Y, Matsumoto S, Matsushita A, Yoshioka M, Shimizu T, Yamahatsu K, Uchida E (2012). Pancreaticojejunostomy with closure of the pancreatic stump by endoscopic linear stapler in laparoscopic pancreaticoduodenectomy: a reliable technique and benefits for pancreatic resection. Asian journal of endoscopic surgery.

[38] Suzuki O, Kondo S, Hirano S, Tanaka E, Kato K, Tsuchikawa T, Yano T, Okamura K, Shichinohe T (2012). Laparoscopic pancreaticoduodenectomy combined with minilaparotomy. Surg Today.

[39] Zeh HJ, Zureikat AH, Secrest A, Dauoudi M, Bartlett D, Moser AJ (2012). Outcomes after robot-assisted pancreaticoduodenectomy for periampullary lesions. Ann Surg Oncol.

[40] Boggi U, Signori S, De Lio N, Perrone VG, Vistoli F, Belluomini M, Cappelli C, Amorese G, Mosca F (2013). Feasibility of robotic pancreaticoduodenectomy. Br J Surg.

[41] Corcione F, Pirozzi F, Cuccurullo D, Piccolboni D, Caracino V, Galante F, Cusano D, Sciuto A (2013). Laparoscopic pancreaticoduodenectomy: experience of 22 cases. Surg Endosc.

[42] Gumbs AA, Croner R, Rodriguez A, Zuker N, Perrakis A, Gayet B (2013). 200 consecutive laparoscopic pancreatic resections performed with a robotically controlled laparoscope holder. Surg Endosc.

[43] Honda G, Kurata M, Okuda Y, Kobayashi S, Sakamoto K, Takahashi K (2013). Laparoscopic pancreaticoduodenectomy: taking advantage of the unique view from the caudal side. J Am Coll Surg.

[44] Jacobs MJ, Kamyab A: Total laparoscopic pancreaticoduodenectomy. JSLS *:* Journal of the Society of Laparoendoscopic Surgeons 2013, 17(2):188–193. 10.4293/108680813X13654754534792 PMC3771783 23925010

[45] Kim SC, Song KB, Jung YS, Kim YH, Park DH, Lee SS, Seo DW, Lee SK, Kim MH, Park KM (2013). Short-term clinical outcomes for 100 consecutive cases of laparoscopic pylorus-preserving pancreatoduodenectomy: improvement with surgical experience. Surg Endosc.

[46] Lee JS, Han JH, Na GH, Choi HJ, Hong TH, You YK, Kim DG (2013). Laparoscopic pancreaticoduodenectomy assisted by mini-laparotomy. Surgical laparoscopy, endoscopy & percutaneous techniques.

[47] Lei Z, Zhifei W, Jun X, Chang L, Lishan X, Yinghui G, Bo Z: Pancreaticojejunostomy sleeve reconstruction after pancreaticoduodenectomy in laparoscopic and open surgery. JSLS *:* Journal of the Society of Laparoendoscopic Surgeons 2013, 17(1):68–73. 10.4293/108680812X13517013318238 PMC3662748 23743374

[48] Machado MA, Makdissi FF, Surjan RC, Machado MC (2013). Laparoscopic pylorus-preserving pancreatoduodenectomy with double jejunal loop reconstruction: an old trick for a new dog. Journal of laparoendoscopic & advanced surgical techniques Part A.

[49] Machado MA, Surjan RC, Goldman SM, Ardengh JC, Makdissi FF (2013). Laparoscopic pancreatic resection. From enucleation to pancreatoduodenectomy. 11-year experience. Arq Gastroenterol.

[50] Mesleh MG, Stauffer JA, Bowers SP, Asbun HJ (2013). Cost analysis of open and laparoscopic pancreaticoduodenectomy: a single institution comparison. Surg Endosc.

[51] Stauffer JA, Raimondo M, Woodward TA, Goldberg RF, Bowers SP, Asbun HJ (2013). Laparoscopic partial sleeve duodenectomy (PSD) for nonampullary duodenal neoplasms: avoiding a whipple by separating the duodenum from the pancreatic head. Pancreas.

[52] Zureikat AH, Moser AJ, Boone BA, Bartlett DL, Zenati M, Zeh HJ (2013). 250 robotic pancreatic resections: safety and feasibility. Ann Surg.

[53] Bao PQ, Mazirka PO, Watkins KT (2014). Retrospective comparison of robot-assisted minimally invasive versus open pancreaticoduodenectomy for periampullary neoplasms. Journal of gastrointestinal surgery : official journal of the Society for Surgery of the Alimentary Tract.

[54] Cho A, Yamamoto H, Kainuma O, Muto Y, Park S, Arimitsu H, Sato M, Souda H, Ikeda A, Nabeya Y (2014). Performing simple and safe dunking pancreaticojejunostomy using mattress sutures in pure laparoscopic pancreaticoduodenectomy. Surg Endosc.

[55] Croome KP, Farnell MB, Que FG, Reid-Lombardo KM, Truty MJ, Nagorney DM, Kendrick ML (2014). Total laparoscopic pancreaticoduodenectomy for pancreatic ductal adenocarcinoma: oncologic advantages over open approaches?. Ann Surg.

[56] Edil BH, Cooper MA, Makary MA (2014). Laparoscopic pancreaticojejunostomy using a barbed suture: a novel technique. Journal of laparoendoscopic & advanced surgical techniques Part A.

[57] Hakeem AR, Verbeke CS, Cairns A, Aldouri A, Smith AM, Menon KV (2014). A matched-pair analysis of laparoscopic versus open pancreaticoduodenectomy: oncological outcomes using Leeds pathology protocol. Hepatobiliary & pancreatic diseases international : HBPD INT.

[58] Hughes SJ, Neichoy B, Behrns KE (2014). Laparoscopic intussuscepting pancreaticojejunostomy. Journal of gastrointestinal surgery : official journal of the Society for Surgery of the Alimentary Tract.

[59] Ji W, Ding K, Kao X, He C, Li N, Li J (2014). Robotic and laparoscopic hybrid pancreaticoduodenectomy: surgical techniques and early outcomes. Chin Med J.

[60] Kuroki T, Kitasato A, Adachi T, Tanaka T, Hirabaru M, Matsushima H, Soyama A, Hidaka M, Takatsuki M, Eguchi S (2014). Learning curve for laparoscopic Pancreaticoduodenectomy: a single Surgeon's experience with consecutive patients. Hepato-Gastroenterology.

[61] Langan RC, Graham JA, Chin AB, Rubinstein AJ, Oza K, Nusbaum JA, Smirniotopoulos J, Kayser R, Jha R, Haddad N (2014). Laparoscopic-assisted versus open pancreaticoduodenectomy: early favorable physical quality-of-life measures. Surgery.

[62] Puntambekar S, Kenawadekar R, Pandit A, Nadkarni A, Bhat NA, Joshi S, Joshi GA (2014). Laparoscopic Supracolic Pancreaticoduodenectomy: a novel technique for complete Uncinate process resection. Hepato-Gastroenterology.

[63] Speicher PJ, Nussbaum DP, White RR, Zani S, Mosca PJ, Blazer DG, 3rd, Clary BM, Pappas TN, Tyler DS, Perez A: Defining the learning curve for team-based laparoscopic pancreaticoduodenectomy. Ann Surg Oncol 2014, 21(12):4014–4019. 10.1245/s10434-014-3839-7 24923222

[64] Wang Y, Bergman S, Piedimonte S, Vanounou T (2014). Bridging the gap between open and minimally invasive pancreaticoduodenectomy: the hybrid approach. Canadian journal of surgery Journal canadien de Chirurgie.

[65] Wellner UF, Kusters S, Sick O, Busch C, Bausch D, Bronsert P, Hopt UT, Karcz KW, Keck T (2014). Hybrid laparoscopic versus open pylorus-preserving pancreatoduodenectomy: retrospective matched case comparison in 80 patients. Langenbeck's Arch Surg.

[66] Adam MA, Choudhury K, Dinan MA, Reed SD, Scheri RP, Blazer DG, Roman SA, Sosa JA (2015). Minimally invasive versus open Pancreaticoduodenectomy for cancer: practice patterns and short-term outcomes among 7061 patients. Ann Surg.

[67] Boone BA, Zenati M, Hogg ME, Steve J, Moser AJ, Bartlett DL, Zeh HJ, Zureikat AH (2015). Assessment of quality outcomes for robotic pancreaticoduodenectomy: identification of the learning curve. JAMA surgery.

[68] Chen S, Chen JZ, Zhan Q, Deng XX, Shen BY, Peng CH, Li HW (2015). Robot-assisted laparoscopic versus open pancreaticoduodenectomy: a prospective, matched, mid-term follow-up study. Surg Endosc.

[69] Croome KP, Farnell MB, Que FG, Reid-Lombardo KM, Truty MJ, Nagorney DM, Kendrick ML (2015). Pancreaticoduodenectomy with major vascular resection: a comparison of laparoscopic versus open approaches. Journal of gastrointestinal surgery : official journal of the Society for Surgery of the Alimentary Tract.

[70] Dokmak S, Fteriche FS, Aussilhou B, Bensafta Y, Levy P, Ruszniewski P, Belghiti J, Sauvanet A (2015). Laparoscopic pancreaticoduodenectomy should not be routine for resection of periampullary tumors. J Am Coll Surg.

[71] Liang S, Jayaraman S (2015). Getting started with minimally invasive Pancreaticoduodenectomy: is it worth it?. Journal of laparoendoscopic & advanced surgical techniques Part A.

[72] Liu Z, MC Y, Zhao R, Liu YF, Zeng JP, Wang XQ, Tan JW (2015). Laparoscopic pancreaticoduodenectomy via a reverse-“V” approach with four ports: initial experience and perioperative outcomes. World J Gastroenterol.

[73] Matsuda M, Haruta S, Shinohara H, Sasaki K, Watanabe G (2015). Pancreaticogastrostomy in pure laparoscopic pancreaticoduodenectomy--a novel pancreatic-gastric anastomosis technique. BMC Surg.

[74] Mendoza AS, Han HS, Yoon YS, Cho JY, Choi Y (2015). Laparoscopy-assisted pancreaticoduodenectomy as minimally invasive surgery for periampullary tumors: a comparison of short-term clinical outcomes of laparoscopy-assisted pancreaticoduodenectomy and open pancreaticoduodenectomy. Journal of hepato-biliary-pancreatic sciences.

[75] Nagakawa Y, Hosokawa Y, Sahara Y, Takishita C, Nakajima T, Hijikata Y, Tago T, Kasuya K, Tsuchida A, Novel A (2015). "Artery first" approach allowing safe resection in laparoscopic Pancreaticoduodenectomy: the Uncinate process first approach. Hepato-Gastroenterology.

[76] Nguyen TK, Zenati MS, Boone BA, Steve J, Hogg ME, Bartlett DL, Zeh HJ, Zureikat AH (2015). Robotic pancreaticoduodenectomy in the presence of aberrant or anomalous hepatic arterial anatomy: safety and oncologic outcomes. HPB : the official journal of the International Hepato Pancreato Biliary Association.

[77] Paniccia A, Schulick RD, Edil BH (2015). Total laparoscopic Pancreaticoduodenectomy: a single-institutional experience. Ann Surg Oncol.

[78] Piedimonte S, Wang Y, Bergman S, Vanounou T (2015). Early experience with robotic pancreatic surgery in a Canadian institution. Canadian journal of surgery Journal canadien de Chirurgie.

[79] Rashid OM, Mullinax JE, Pimiento JM, Meredith KL, Malafa MP (2015). Robotic Whipple procedure for pancreatic cancer: the Moffitt Cancer Center pathway. Cancer control : journal of the Moffitt Cancer Center.

[80] Senthilnathan P, Srivatsan Gurumurthy S, Gul SI, Sabnis S, Natesan AV, Palanisamy NV, Praveen Raj P, Subbiah R, Ramakrishnan P, Palanivelu C (2015). Long-term results of laparoscopic pancreaticoduodenectomy for pancreatic and periampullary cancer-experience of 130 cases from a tertiary-care center in South India. Journal of laparoendoscopic & advanced surgical techniques Part A.

[81] Sharpe SM, Talamonti MS, Wang CE, Prinz RA, Roggin KK, Bentrem DJ, Winchester DJ, Marsh RD, Stocker SJ, Baker MS (2015). Early National Experience with laparoscopic Pancreaticoduodenectomy for ductal adenocarcinoma: a comparison of laparoscopic Pancreaticoduodenectomy and open Pancreaticoduodenectomy from the National Cancer Data Base. J Am Coll Surg.

[82] Shubert CR, Wagie AE, Farnell MB, Nagorney DM, Que FG, Reid Lombardo KM, Truty MJ, Smoot RL, Kendrick ML (2015). Clinical risk score to predict pancreatic fistula after Pancreatoduodenectomy: independent external validation for open and laparoscopic approaches. J Am Coll Surg.

[83] Song KB, Kim SC, Hwang DW, Lee JH, Lee DJ, Lee JW, Park KM, Lee YJ (2015). Matched case-control analysis comparing laparoscopic and open pylorus-preserving Pancreaticoduodenectomy in patients with Periampullary tumors. Ann Surg.

[84] Tan CL, Zhang H, Peng B, Li KZ (2015). Outcome and costs of laparoscopic pancreaticoduodenectomy during the initial learning curve vs laparotomy. World J Gastroenterol.

[85] Tee MC, Croome KP, Shubert CR, Farnell MB, Truty MJ, Que FG, Reid-Lombardo KM, Smoot RL, Nagorney DM, Kendrick ML (2015). Laparoscopic pancreatoduodenectomy does not completely mitigate increased perioperative risks in elderly patients. HPB : the official journal of the International Hepato Pancreato Biliary Association.

[86] Wang M, Zhang H, Wu Z, Zhang Z, Peng B (2015). Laparoscopic pancreaticoduodenectomy: single-surgeon experience. Surg Endosc.

[87] Adam MA, Thomas S, Youngwirth L, Pappas T, Roman SA, Sosa JA. Defining a hospital volume threshold for minimally invasive Pancreaticoduodenectomy in the United States. JAMA surgery. 2016; 10.1001/jamasurg.2016.4753 PMC5470427 28030713

[88] Baker EH, Ross SW, Seshadri R, Swan RZ, Iannitti DA, Vrochides D, Martinie JB (2016). Robotic pancreaticoduodenectomy: comparison of complications and cost to the open approach. The international journal of medical robotics + computer assisted surgery : MRCAS.

[89] Battal M, Yilmaz A, Ozturk G, Karatepe O (2016). The difficulties encountered in conversion from classic pancreaticoduodenectomy to total laparoscopic pancreaticoduodenectomy. Journal of minimal access surgery.

[90] Coratti A, Di Marino M, Coratti F, Baldoni G, Guerra F, Amore Bonapasta S, Bencini L, Farsi M, Annecchiarico M (2016). Initial experience with robotic pancreatic surgery: technical feasibility and oncological implications. Surgical laparoscopy, endoscopy & percutaneous techniques.

[91] Cunningham KE, Zenati MS, Petrie JR, Steve JL, Hogg ME, Zeh HJ, 3rd, Zureikat AH: A policy of omitting an intensive care unit stay after robotic pancreaticoduodenectomy is safe and cost-effective. J Surg Res 2016, 204(1):8–14. 10.1016/j.jss.2016.04.023 27451861

[92] Delitto D, Luckhurst CM, Black BS, Beck JL, George TJ, Sarosi GA, Thomas RM, Trevino JG, Behrns KE, Hughes SJ (2016). Oncologic and perioperative outcomes following selective application of laparoscopic Pancreaticoduodenectomy for Periampullary malignancies. J Gastrointest Surg.

[93] Fan Y, Zhao YH, Pang L, Kang YX, Kang BX, Liu YY, Fu J, Xia BW, Wang C, Zhang YC. Successful experience of laparoscopic Pancreaticoduodenectomy and digestive tract reconstruction with minimized complications rate by 14 case reports. Medicine. 2016;95(17) 10.1097/MD.0000000000003167 PMC4998677 27124014

[94] Girgis MD, Zenati MS, Steve J, Bartlett DL, Zureikat A, Zeh HJ, Hogg ME. Robotic approach mitigates perioperative morbidity in obese patients following pancreaticoduodenectomy. HPB : the official journal of the International Hepato Pancreato Biliary Association. 2016; 10.1016/j.hpb.2016.11.008 28038966

[95] Hsu CW, Lin LF, Law MK (2016). Purse-string suture without pancreatic parenchymal stitches in pancreaticojejunostomy during laparoscopic pancreaticoduodenectomy. Surg Pract.

[96] Kantor O, Talamonti MS, Sharpe S, Lutfi W, Winchester DJ, Roggin KK, Bentrem DJ, Prinz RA, Baker MS. Laparoscopic pancreaticoduodenectomy for adenocarcinoma provides short-term oncologic outcomes and long-term overall survival rates similar to those for open pancreaticoduodenectomy. Am J Surg. 2016; 10.1016/j.amjsurg.2016.10.030 28049562

[97] Kauffmann EF, Napoli N, Menonna F, Vistoli F, Amorese G, Campani D, Pollina LE, Funel N, Cappelli C, Caramella D (2016). Robotic pancreatoduodenectomy with vascular resection. Langenbeck's Arch Surg.

[98] Kim EY, Hong TH (2016). Total laparoscopic Pancreaticoduodenectomy using a new technique of Pancreaticojejunostomy with two Transpancreatic sutures with buttresses. Journal of laparoendoscopic & advanced surgical techniques Part A.

[99] Kutlu OC, Lee JE, Katz MH, Tzeng CD, Wolff RA, Varadhachary GR, Vauthey JN, Fleming JB, Conrad C. Open Pancreaticoduodenectomy case volume predicts outcome of laparoscopic approach: a population-based analysis. Ann Surg. 2016; 10.1097/SLA.0000000000002111 28045744

[100] Liao CH, Liu YY, Wang SY, Liu KH, Yeh CN, Yeh TS. The feasibility of laparoscopic pancreaticoduodenectomy-a stepwise procedure and learning curve. Langenbeck's Arch Surg. 2016; 10.1007/s00423-016-1541-x 27987099

[101] Liu R, Zhang T, Zhao ZM, Tan XL, Zhao GD, Zhang X, Xu Y. The surgical outcomes of robot-assisted laparoscopic pancreaticoduodenectomy versus laparoscopic pancreaticoduodenectomy for periampullary neoplasms: a comparative study of a single center. Surg Endosc. 2016; 10.1007/s00464-016-5238-6 27631318

[102] Machado MA, Surjan RC, Basseres T, Silva IB, Makdissi FF (2016). Laparoscopic Pancreatoduodenectomy in 50 consecutive patients with no mortality: a single-center experience. Journal of laparoendoscopic & advanced surgical techniques Part A.

[103] Napoli N, Kauffmann EF, Menonna F, Perrone VG, Brozzetti S, Boggi U (2016). Indications, technique, and results of robotic pancreatoduodenectomy. Updat Surg.

[104] Nussbaum DP, Adam MA, Youngwirth LM, Ganapathi AM, Roman SA, Tyler DS, Sosa JA, Blazer DG (2016). Minimally invasive Pancreaticoduodenectomy does not improve use or time to initiation of adjuvant chemotherapy for patients with pancreatic adenocarcinoma. Ann Surg Oncol.

[105] Polanco PM, Zenati MS, Hogg ME, Shakir M, Boone BA, Bartlett DL, Zeh HJ, Zureikat AH (2016). An analysis of risk factors for pancreatic fistula after robotic pancreaticoduodenectomy: outcomes from a consecutive series of standardized pancreatic reconstructions. Surg Endosc.

[106] Poves I, Morato O, Burdio F, Grande L. Laparoscopic-adapted Blumgart pancreaticojejunostomy in laparoscopic pancreaticoduodenectomy. Surg Endosc. 2016; 10.1007/s00464-016-5294-y 27804043

[107] Stauffer JA, Coppola A, Villacreses D, Mody K, Johnson E, Li Z, Asbun HJ. Laparoscopic versus open pancreaticoduodenectomy for pancreatic adenocarcinoma: long-term results at a single institution. Surg Endosc. 2016; 10.1007/s00464-016-5222-1 27604369

[108] Tran TB, Dua MM, Worhunsky DJ, Poultsides GA, Norton JA, Visser BC (2016). The first decade of laparoscopic Pancreaticoduodenectomy in the United States: costs and outcomes using the Nationwide inpatient sample. Surg Endosc.

[109] Wang MJ, Meng LW, Cai YQ, Li YB, Wang X, Zhang ZD, Peng B (2016). Learning curve for laparoscopic Pancreaticoduodenectomy: a CUSUM analysis. J Gastrointest Surg.

[110] Zureikat AH, Postlewait LM, Liu Y, Gillespie TW, Weber SM, Abbott DE, Ahmad SA, Maithel SK, Hogg ME, Zenati M (2016). A multi-institutional comparison of perioperative outcomes of robotic and open Pancreaticoduodenectomy. Ann Surg.

[111] Wang M, Xu S, Zhang H, Peng S, Zhu F, Qin R. Imbedding pancreaticojejunostomy used in pure laparoscopic pancreaticoduodenectomy for nondilated pancreatic duct. Surg Endosc. 2017; 10.1007/s00464-016-4805-1 28078460

[112] Elberm H, Ravikumar R, Sabin C, Abu Hilal M, Al-Hilli A, Aroori S, Bond-Smith G, Bramhall S, Coldham C, Hammond J (2015). Outcome after pancreaticoduodenectomy for T3 adenocarcinoma: a multivariable analysis from the UK vascular resection for pancreatic cancer study group. European journal of surgical oncology : the journal of the European Society of Surgical Oncology and the British Association of Surgical Oncology.

[113] de Wilde RF, Besselink MG, van der Tweel I, de Hingh IH, van Eijck CH, Dejong CH, Porte RJ, Gouma DJ, Busch OR, Molenaar IQ (2012). Impact of nationwide centralization of pancreaticoduodenectomy on hospital mortality. Br J Surg.

[114] Topal B, Van de Sande S, Fieuws S, Penninckx F (2007). Effect of centralization of pancreaticoduodenectomy on nationwide hospital mortality and length of stay. Br J Surg.

[115] Xiong JJ, Tan CL, Szatmary P, Huang W, Ke NW, WM H, Nunes QM, Sutton R, Liu XB (2014). Meta-analysis of pancreaticogastrostomy versus pancreaticojejunostomy after pancreaticoduodenectomy. Br J Surg.

[116] Ramana CV, DeBerge MP, Kumar A, Alia CS, Durbin JE, Enelow RI (2015). Inflammatory impact of IFN-gamma in CD8+ T cell-mediated lung injury is mediated by both Stat1-dependent and -independent pathways. American journal of physiology Lung cellular and molecular physiology.

[117] Fuks D, Cauchy F, Fteriche S, Nomi T, Schwarz L, Dokmak S, Scatton O, Fusco G, Belghiti J, Gayet B (2016). Laparoscopy decreases pulmonary complications in patients undergoing major liver resection: a propensity score analysis. Ann Surg.

[118] Li H, Peng B (2015). Total laparoscopic pancreaticoduodenectomy may benefit patients with severe impaired pulmonary function. Surgical laparoscopy, endoscopy & percutaneous techniques.

[119] Gagner M, Palermo M (2009). Laparoscopic Whipple procedure: review of the literature. J Hepato-Biliary-Pancreat Surg.

[120] Hartwig W, Werner J, Jager D, Debus J, Buchler MW (2013). Improvement of surgical results for pancreatic cancer. The Lancet Oncology.

[121] Ryan DP, Hong TS, Bardeesy N (2014). Pancreatic adenocarcinoma. N Engl J Med.

