# Supplementary figures and images for: Minimally invasive pancreaticoduodenectomy for periampullary disease: a comprehensive review of literature and meta-analysis of outcomes compared with open surgery
Source: BMC Gastroenterol. 2017 Nov 23;17:120. doi: 10.1186/s12876-017-0691-9 (PMC5701376; doi:10.1186/s12876-017-0691-9)

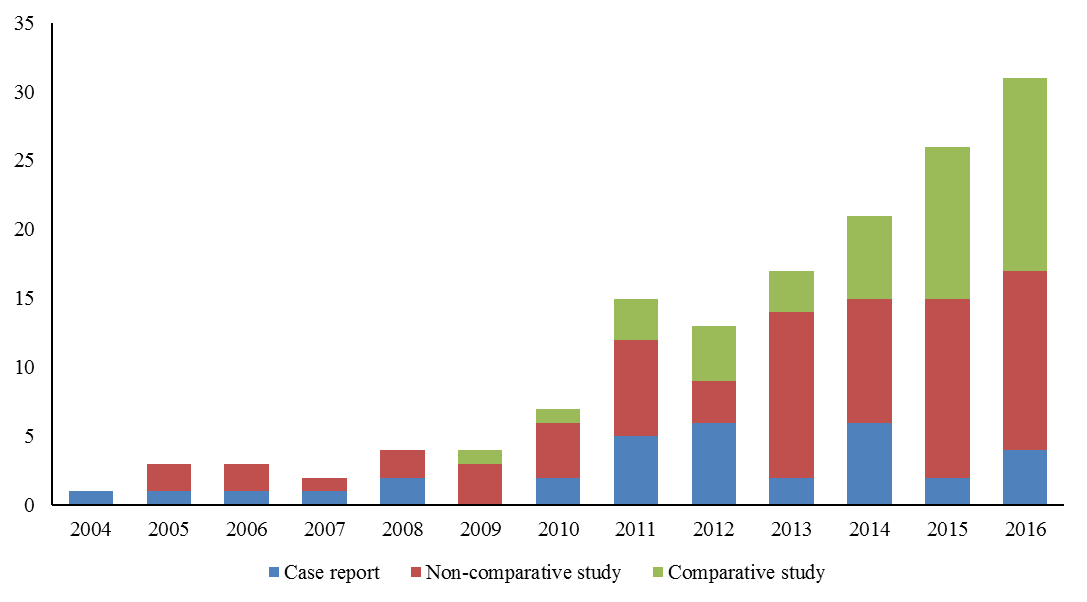

Supplement: Supplementary file 1 — Number of original publications concerning MIPD by year according to the study design (abstracts, letters, comments, reviews were not included). (TIFF 56 kb) [file 12876_2017_691_MOESM1_ESM.tif]

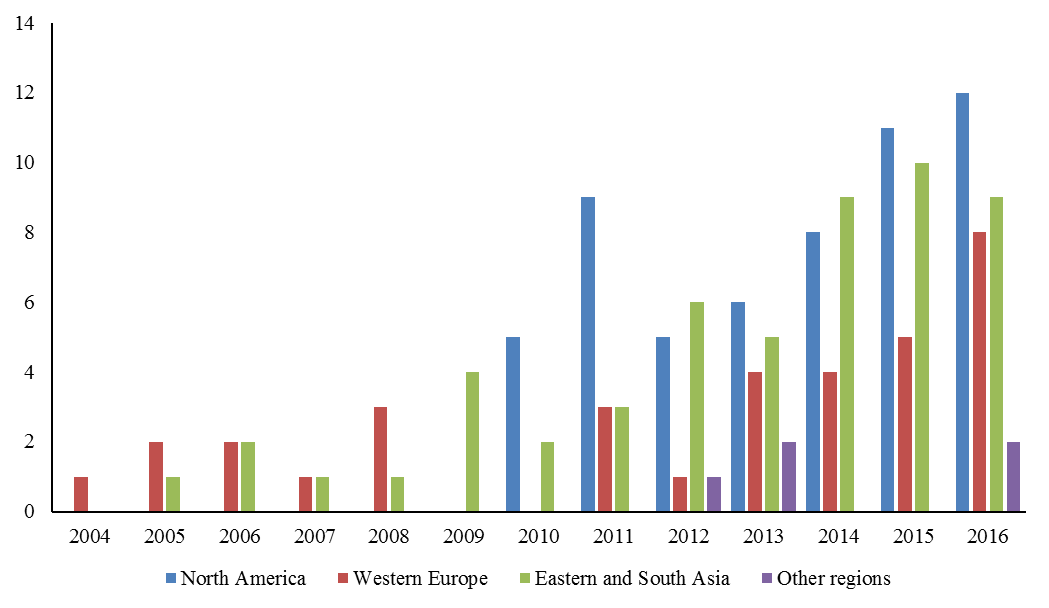

Supplement: Supplementary file 2 — Number of total publications concerning MIPD by year according to the place of origin (the majority North American studies came from USA; other regions included Turkey and Brazil). (TIFF 67 kb) [file 12876_2017_691_MOESM2_ESM.tif]
