# Supplementary material for: Minimally invasive pancreaticoduodenectomy for periampullary disease: a comprehensive review of literature and meta-analysis of outcomes compared with open surgery
Source: BMC Gastroenterol. 2017 Nov 23;17:120. doi: 10.1186/s12876-017-0691-9 (PMC5701376; doi:10.1186/s12876-017-0691-9)
Supplement: Supplementary file 3 — Published articles of MIPD. (DOCX 52 kb) [file 12876_2017_691_MOESM3_ESM.docx]

**Additional file 3** Published articles of MIPD.

| **Author** | **Region** | **Facility** | **Journal** | **Design** | **Year** | **Period** | **No.** | **Summary** |
| --- | --- | --- | --- | --- | --- | --- | --- | --- |
| Dulucq [12] | France | ILS, Bordeaux | Surg Endosc | Case series | 2005 | 1995-2003 | 11 | Clinical experience of LAPD |
| Staudacher [13] | Italy | San Raﬀaele Scientiﬁc Institute, Milan | Surg Endosc | Case series | 2005 | 2003-2004 | 4 | Clinical experience of LAPD |
| Dulucq [14] | France | ILS, Bordeaux | Surg Endosc | Case series | 2006 | 1999-2005 | 22 | Comparison of totally laparoscopic and laparoscopic assisted PD |
| Palanivelu [15] | India | Gem Hospital, Coimbatore | J Am CollSurg | Case series | 2007 | 1998-2006 | 45 | Clinical experience and long-term survival outcomes |
| Pugliese [16] | Italy | Niguarda Hospital, Milan | Surg Laparosc Endosc Percutan Tech | Case series | 2008 | 2002-2006 | 19 | Clinical experiences and long-term survival outcomes |
| Cho [17] | Japan | Chiba Cancer Center Hospital | Am J Surg | Cohort study | 2009 | 2007-2008 | 15 | Comparison of short-term outcomes of LAPPPD and OPPPD |
| Shinohara [19] | Japan | Fujita Health University School of Medicine, Toyoake | Langenbecks Arch Surg | Case series | 2009 | 2008 | 5 | Description of TLPD for advanced gastric cancer |
| Palanivelu [18] | India | GEM Hospital, Ramnathapuram, Coimbatore | J Hepatobiliary Pancreat Surg | Case series | 2009 | 1998-2009 | 75 | A decade long experience of LPD with short-term outcomes |
| Kendrick [22] | USA | Mayo Clinic, Rochester | Arch Surg | Case series | 2010 | 2007-2009 | 62 | Early experiences of TLPD |
| Narula [24] | USA | Ohio State University Medical Center, Columbus | Pancreas | Case series | 2010 | 2006-2007 | 5 | Initial experience of hybrid PD, short- and mid-term outcomes |
| Giulianotti [21] | USA, Italy | Misericordia Hospital, Grosseto; University of Illinois Medical Center, Chicago | Surg Endosc | Case series | 2010 | 2000- 2009 | 50 | Single-surgeon experience in two institutions |
| Kuroki [23] | Japan | Graduate School of Biomedical Sciences, Nagasaki University | Surg Endosc | Case series | 2010 | 2008-2009 | 9 | Technique report of a new LPD method |
| Buchs [20] | USA | University of Illinois Medical Center, Chicago | World J Surg | Cohort study | 2010 | 2007-2010 | 41 | Investigation of RAPD for elderly patients |
| Zureikat [32] | USA | University of Pittsburgh Medical Center | Arch Surg | Case series | 2011 | 2008-2010 | 24 | Initial experience of LAPD, short-term outcomes |
| Kendrick [20] | USA | Mayo Clinic, Rochester | HPB (Oxford) | Case series | 2011 | 2007-2010 | 11 | Initial experience of TLPD with major venous resection |
| Zhou [30] | China | General Hospital of PLA Second Artillery, Beijing | Int J Med Robot | Cohort study | 2011 | 2009 | 8 | Comparison of short-term outcomes of RPD and OPD |
| Zureikat [31] | USA | University of Pittsburgh Medical Center | J Gastrointest Surg | Case match | 2011 | 2008-2010 | 14 | Comparison of short- and mid-term outcomes of LPD and OPD |
| Horiguchi [28] | Japan | Fujita Health University, Toyoake | J Hepatobiliary Pancreat Sci | Case series | 2011 | 2009-2010 | 3 | Technique report of TLPD with clinical experience |
| Ammori [25] | UK | North Manchester General Hospital | Surg Endosc | Case series | 2011 | 2002-2008 | 7 | Clinical experience of LPD with literature review |
| Buchs [26] | USA | University of Illinois Medical Center, Chicago | World J Surg | Cohort study | 2011 | 2002-2010 | 44 | Comparison of short-term outcomes of RPD and OPD |
| Giulianotti [27] | USA | University of Illinois at Chicago | Pancreas | Case series | 2011 | 2007-2010 | 2 | Experiences of RPD with vascular resection for locally advanced pancreatic tumors |
| Zeh [39] | USA | University of Pittsburgh Medical Center | Ann Surg Oncol | Case series | 2012 | 2008-2010 | 50 | Early experiences of RAPD |
| Nakamura [37] | Japan | Nippon Medical School, Tokyo | Asian J Endosc Surg | Case series | 2012 | 2011 | 12 | Technique report of PJ in LPD |
| Kuroki [35] | Japan | Nagasaki University Hospital | Hepatogastroenterology | Cohort study | 2012 | 2008-2010 | 20 | Comparison of short-term outcomes of LAPD and OPD |
| Lai [36] | China | Pamela Youde Nethersole Eastern Hospital, Hong Kong | Int J Surg | Cohort study | 2012 | 2000-2012 | 20 | Comparison of short-term outcomes of RAPD and OPD |
| Asbun [33] | USA | Mayo Clinic, Jacksonville | J Am Coll Surg | Cohort study | 2012 | 2005-2011 | 53 | Comparison of short-term outcomes of LPD and OPD |
| Chalikonda [34] | USA | Cleveland Clinic | Surg Endosc | Case match | 2012 | 2009-2010 | 30 | Comparison of short-term outcomes of RAPD and OPD |
| Suzuki [38] | Japan | Hokkaido University Graduate School of Medicine, Sapporo | Surg Today | Case series | 2012 | 2006-2008 | 6 | Clinical experience of LPD with mini-laparotomy |
| Zureikat [52] | USA | University of Pittsburgh Medical Center | Ann Surg | Case series | 2013 | 2008-2012 | 132 | Clinical experience of RPD and short-term outcomes |
| Machado [49] | Brasil | Hospital Sírio Libanês, São Paulo | Arq Gastroenterol | Case series | 2013 | 2001-2012 | 7 | Clinical experience of LPPPD |
| Boggi [40] | Italy | Pisa University Hospital | Br J Surg | Case series | 2013 | 2008-2011 | 34 | Clinical experience of RPD with technique detail and short-term outcomes |
| Honda [43] | Japan | Tokyo Metropolitan Cancer and Infectious Diseases Center | J Am Coll Surg | Case series | 2013 | 2011-2013 | 26 | Technique report dissecting pancreatic head from the mesenteric vessels |
| Jacobs [44] | USA | St. John Providence Health, Southfield | JSLS | Case series | 2013 | 2011-2012 | 5 | Early experiences of TLPD |
| Lei [47] | China | Fourth Affiliated Hospital of Harbin Medical University | JSLS | Cohort study | 2013 | 2004-2010 | 11 | Comparison of short-term outcomes of LPD and OPD and assessment of a PJ method |
| Mesleh [50] | USA | Mayo Clinic, Jacksonville | Surg Endosc | Cohort study | 2013 | 2009-2012 | 75 | Comparison of short-term outcomes of LPD and OPD, cost analysis |
| Gumbs [42] | USA | Summit Medical Group, Berkeley Heights | Surg Endosc | Case series | 2013 | 1994-2011 | 72 | Clinical experience of RPD with literature review |
| Corcione [41] | Italy | Azienda Ospedalieradei Colli-Monaldi Hospital, Naples | Surg Endosc | Case series | 2013 | 2003-2010 | 22 | Clinical experience and short-term outcomes of LPD |
| Kim [45] | Korea | University of Ulsan College of Medicine and Asan Medical Center, Seoul | Surg Endosc | Case series | 2013 | 2007-2011 | 100 | Clinical experience and short-term outcomes of LPPPD |
| Lee [46] | Korea | Armed Forces Capital Hospital, Seongnam | Surg Laparosc Endosc Percutan Tech | Case series | 2013 | 2009-2012 | 42 | Clinical experience of LPD with mini-laparotomy |
| Stauffer [51] | USA | Mayo Clinic, Jacksonville | Pancreas | Case series | 2013 | 2008-2011 | 10 | Technique report of laparoscopic partial sleeve duodenectomy |
| Machado [48] | Brazil | University of São Paulo | J Laparoendosc Adv Surg Tech A | Case series | 2013 | NR | 3 | Investigation of double jejunal loop reconstruction for LPPPD |
| Speicher [63] | USA | Duke University Medical Center, Durham | Ann Surg Oncol | Cohort study | 2013 | 2010-2013 | 56 | Investigation of learning curves for LPD and comparison of short-term outcomes with OPD |
| Croome [55] | USA | Mayo Clinic, Rochester | Ann Surg | Cohort study | 2014 | 2008-2013 | 108 | Comparison of short-term surgical and long-term survival outcomes of TLPD and OPD |
| Wang [64] | Canada | Davis Jewish General Hospital, Montréal | Can J Surg | Cohort study | 2014 | 2009-2013 | 13 | Comparison of short-term outcomes of LPD and OPD |
| Wu [59] | China | Jinling Hospital, Nanjing | Chin Med J (Engl) | Case series | 2014 | 2010-2012 | 6 | Initial experience of LPD and RPD |
| Hakeem [57] | UK | St James's University Hospital, Leeds | Hepatobiliary Pancreat Dis Int | Case match | 2014 | 2005-2009 | 12 | Comparison of short-term surgical and long-term survival outcomes of LPD and OPD |
| Bao [53] | USA | Stony Brook University Medical Center | J Gastrointest Surg | Case match | 2014 | 2009-2011 | 28 | Comparison of short-term surgical outcomes of RPD and OPD |
| Edil [56] | USA | Johns Hopkins University School of Medicine, Baltimore | J Laparoendosc Adv Surg Tech A | Case series | 2014 | 2010-2012 | 19 | Technique report of intracorporeal hand-sewn PJ in TLPD |
| Wellner [65] | Germany | University Medical Centers Freiburg and Lübeck | Langenbecks Arch Surg | Case match | 2014 | 2010-2013 | 40 | Comparison of short-term surgical outcomes of LPPPD and OPPPD |
| Cho [54] | Japan | Chiba Cancer Center Hospital | Surg Endosc | Case series | 2014 | 2011-2012 | 15 | Technique report of intracorporeal hand-sewn PJ in TLPD |
| Langan [61] | USA | Georgetown University Hospital, Washington | Surgery | Cohort study | 2014 | 2010-2013 | 28 | Comparison of short-term surgical outcomes of LPD and OPD, investigation of QOL |
| Hughes [58] | USA | University of Florida College of Medicine, Gainesville | J Gastrointest Surg | Case series | 2014 | 2012 | 24 | Technique report of intracorporeal hand-sewn end-to-end intussuscepting PJ in TLPD |
| Kuroki [60] | Japan | Nagasaki University Hospital | Hepatogastroenterology | Case series | 2014 | 2008-2012 | 30 | Investigation of learning curves for LPD and short-term outcomes |
| Puntambekar [62] | India | Galaxy Care Laparoscopy Institute, Pune, Maharashtra | Hepatogastroenterology | Case series | 2014 | 2008-2012 | 38 | Clinical experience of LPD with literature review focusing on complete uncinate process resection |
| Song [83] | Korea | Asan Medical Center, Seoul | Ann Surg | Cohort study | 2015 | 2007-2012 | 97 | Comparison of short- and long-term outcomes of LPPPD and OPPPD |
| Adam [66] | USA | The National Cancer Database (NCDB) | Ann Surg | Cohort study | 2015 | 2010-2011 | 983 | Description of national practice to MIPD and compare short-term outcomes with OPD |
| Paniccia [77] | USA | University of Colorado Anschutz Medical Campus, Aurora | Ann Surg Oncol | Case series | 2015 | 2013-2014 | 30 | Clinical experience of TLPD and short-term outcomes |
| Matsuda [73] | Japan | Toranomon Hospital, Tokyo | BMC Surg | Case series | 2015 | 2012-2013 | 5 | Technique report of intracorporeal hand-sewn PG in TLPD |
| Rashid [79] | USA | Moffitt Cancer Center, Tampa | Cancer Control | Case series | 2015 | 2012-2013 | 14 | Early experiences of RPD |
| Piedimonte [78] | Canada | Davis Jewish General Hospital, Montréal | Can J Surg | Case series | 2015 | 2010-2014 | 26 | Early experiences of RAPD and LAPD |
| Tee [85] | USA | Mayo Clinic, Rochester | HPB (Oxford) | Cohort study | 2015 | 2007-2014 | 113 | Investigation of TLPD for elderly patients |
| Nguyen [76] | USA | University of Pittsburgh Medical Center | HPB (Oxford) | Cohort study | 2015 | 2008-2013 | 142 | Investigation of the influence of hepatic arterial anomalies in RPD |
| Boone [67] | USA | University of Pittsburgh Medical Center | JAMA Surg | Case series | 2015 | 2008-2014 | 200 | Investigation of learning curves for RPD |
| Dokmak [70] | France | Beaujon Hospital, Paris | J Am Coll Surg | Case match | 2015 | 2011-2014 | 46 | Comparison of short-term outcomes of LPD and OPD, investigation of learning curves |
| Sharpe [81] | USA | The National Cancer Database (NCDB) | J Am Coll Surg | Cohort study | 2015 | 2010-2011 | 384 | Early national experience with LPD and comparison with OPD |
| Croome [69] | USA | Mayo Clinic, Rochester | J Gastrointest Surg | Cohort study | 2015 | 2007-2013 | 31 | Comparison of short-term outcomes of TLPD and OPD with major vascular resection |
| Mendoza [74] | Korea | Seoul National University Bundang Hospital | J Hepatobiliary Pancreat Sci | Cohort study | 2015 | 2014 | 18 | Comparison of short-term outcomes of LAPD and OPD |
| Liang [71] | Canada | St. Joseph’s Health Centre, Toronto | J Laparoendosc Adv Surg Tech A | Cohort study | 2015 | 2011-2013 | 15 | Comparison of short-term outcomes of LPD and OPD, cost analysis |
| Senthilnathan [80] | India | Gem Hospital and Research Centre, Coimbatore | J Laparoendosc Adv Surg Tech A | Case series | 2015 | 1998-2013 | 130 | Short-term surgical and long-term survival outcomes of LPD |
| Chen [68] | China | Ruijin Hospital, Shanghai | Surg Endosc | Case match | 2015 | 2010-2013 | 60 | Comparison of short- and mid-term outcomes of RAPD and OPD |
| Wang [86] | China | West China Hospital, Chengdu | Surg Endosc | Case series | 2015 | 2010-2013 | 31 | Clinical experience of LPD, short- and mid-term outcomes |
| Tan [84] | China | West China Hospital, Chengdu | World J Gastroenterol | Case match | 2015 | 2009-2014 | 30 | Comparison of short-term outcomes of TLPD and OPD, cost analysis |
| Liu [72] | China | Jinan Central Hospital, Shandong University | World J Gastroenterol | Case series | 2015 | 2011-2012 | 21 | Initial experience of LPD using a new approach of ports |
| Shubert [82] | USA | Mayo Clinic, Rochester | J Am Coll Surg | Case series | 2015 | 2007-2014 | 269 | Evaluation of the clinical risk score to predict pancreatic fistula after LPD |
| Nagakawa [75] | Japan | Tokyo Medical University | Hepatogastroenterology | Case series | 2015 | NR | 10 | Evaluation of a new approach uncinate process resection in LPD |
| Nussbaum [104] | USA | The National Cancer Database (NCDB) | Ann SurgOncol | Cohort study | 2016 | 2010-2012 | 1191 | Investigation of the MIPD influence in initiation of adjuvant chemotherapy |
| Kim [98] | Korea | Seoul St. Mary's Hospital | J LaparoendoscAdvSurg Tech A | Case series | 2016 | 2013-2015 | 12 | Technique report of intracorporeal hand-sewn PJ in TLPD |
| Polanco [105] | USA | University of PittsburghMedical Center | Surg Endosc | Case series | 2016 | 2008-2013 | 150 | Analysis of risk factors for pancreatic ﬁstula after RPD |
| Coratti [90] | Italy | Careggi University Hospital, Florence | Surg Laparosc Endosc Percutan Tech | Case series | 2016 | 2010 | 36 | Initial experience of RAPD, short- and long-term oncological outcomes |
| Battal [89] | Turkey | SisliEtfal Research Hospital, Istanbul | J Minim Access Surg | Case series | 2016 | 2011-2014 | 22 | Initial experience of TLPD |
| Delitto [92] | USA | University of Florida Health Science Center, Gainesville | J Gastrointest Surg | Cohort study | 2016 | 2010-2014 | 52 | Comparison of short- and long-term outcomes of RAPD and OPD |
| Fan [93] | China | The Second Hospital of Lanzhou University | Medicine (Baltimore) | Case series | 2016 | 2013-2015 | 14 | Initial experience of TLPD |
| Hsu [95] | Taiwan | Tungs’ Taichung Metro Harbor Hospital, Taichung | Surg Pract | Case series | 2016 | 2013-2014 | 5 | Technique report of intracorporeal purse-string suture PJ in TLPD |
| Machado [102] | Brazil | University of São Paulo, Sírio Libanês Hospital | J Laparoendosc Adv Surg Tech A | Case series | 2016 | 2012-2015 | 50 | Clinical experiences and short-term survival outcomes |
| Poves [106] | Spain | Hospital del Mar, Autonomous University of Barcelona | Surg Endosc | Cohort study | 2016 | 2013-2016 | 13 | Technique report of laparoscopic-adapted Blumgart PJ with comparison of short-term outcomes to OPD |
| Tran [108] | USA | Nation-wide Inpatient Sample (NIS) | Surg Endosc | Cohort study | 2016 | 2000-2010 | 681 | The first decade of national experience with LPD and comparison with OPD, cost analysis |
| Wang [109] | China | West China Hospital,Chengdu | J Gastrointest Surg | Case series | 2016 | 2010-20115 | 57 | Investigation of learning curves for LPD and short-term outcomes |
| Zureikat [110] | USA | University of Pittsburgh Medical Center; Cleveland Clinic Foundation | Ann Surg | Cohort study | 2016 | 2011-2015 | 211 | A multi-institutional comparison of perioperative outcomes of RPD and OPD |
| Kantor [96] | USA | The National Cancer Database (NCDB) | Am J Surg | Cohort study | 2016 | 2010-2013 | 828 | National data for comparison of LPD and OPD for PDAC with short- and long-term outcomes |
| Kutlu [99] | USA | The National Cancer Database (NCDB) | Ann Surg | Cohort study | 2016 | 2010-2011 | 430 | Identification of differences in LPD outcomes between low-volume and high-volume center |
| Girgis [94] | USA | University of Pittsburgh Medical Center | HPB (Oxford) | Cohort study | 2016 | 2011-2015 | 70 | Evaluation of the inﬂuence of obesity on the surgical outcomes of RPD |
| Liao [100] | Taiwan | Chang Gung Memorial Hospital, Taoyuan | Langenbecks Arch Surg | Case series | 2016 | 2012-2015 | 12 | Initial experience of LAPD, short-term outcomes |
| Liu [101] | China | Chinese PLA General Hospital, Beijing | Surg Endosc | Cohort study | 2016 | 2015-2016 | 52 | Comparison of short-term outcomes of RPD and LPD |
| Stauffer [107] | USA | Mayo Clinic, Jacksonville | Surg Endosc | Cohort study | 2016 | 1995-2014 | 58 | Comparison of short and long-term outcomes of LPD and OPD for PDAC |
| Baker [88] | USA | Carolinas Medical Center, Charlotte | Int J Med Robot | Cohort study | 2016 | 2012-2013 | 22 | Comparison of short-term outcomes of RPD and OPD with cost analysis |
| Adam [87] | USA | the Health Care Utilization Project National Inpatient Sample data sets (HCUP-NIS) | JAMA Surg | Cohort study | 2016 | 2000-2012 | 865 | Comparison of the outcomes for MIPD between low-volume hospital and high-volume center |
| Napoli [103] | Italy | University of Pisa | Updates Surg | Case series | 2016 | 2009-2014 | 112 | Single-center, large-sample, short-term outcomes of RPD |
| Kauffmann [97] | Italy | University of Pisa | Langenbecks Arch Surg | Cohort study | 2016 | 2008-2014 | 116 | Evaluation of vascular resection of RPD with a systematic review of the literature |
| Cunningham [91] | USA | Pittsburgh Medical Center | J Surg Res | Cohort study | 2016 | 2014-2015 | 96 | Evaluation of necessity of intensive care unit stay after RPD |
| Wang [111] | China | Affiliated Tongji Hospital, Tongji Medical College, Wuhan | Surg Endosc | Case series | 2017 | 2014-2015 | 52 | Technique report of pure laparoscopic imbedding PJ for nondilated pancreatic duct |
