# Supplementary material for: Minimally invasive pancreaticoduodenectomy for periampullary disease: a comprehensive review of literature and meta-analysis of outcomes compared with open surgery
Source: BMC Gastroenterol. 2017 Nov 23;17:120. doi: 10.1186/s12876-017-0691-9 (PMC5701376; doi:10.1186/s12876-017-0691-9)
Supplement: Supplementary file 4 — Baseline and quality assessment based on the NOS for meta-analysis included studies. (DOCX 30 kb) [file 12876_2017_691_MOESM4_ESM.docx]

**Additional file 4** Baseline and quality assessment based on the NOS for meta-analysis included studies.

| **Author** | **Sample size** | | **ITT** | **ISGPF** | **Matched**  **factors** | **Selection (Out of 4)** | | | | **Comparability**  **(Out of 2)** | **Outcomes (Out of 3)** | | | **Total** |
| --- | --- | --- | --- | --- | --- | --- | --- | --- | --- | --- | --- | --- | --- | --- |
|  | **MIPD** | **OPD** |  |  |  | **①** | **②** | **③** | **④** |  | **⑤** | **⑥** | **⑦** |  |
| Cho [17] | 15 | 15 | unclear | Yes | —— | * | * | * | * | * | * |  |  | 6 |
| Zhou [30] | 8 | 8 | unclear | No | —— | * | * | * | * | * | * |  |  | 6 |
| Zureikat [31] | 14 | 14 | No | Yes | abcef | * | * | * | * | ** | * |  |  | 7 |
| Buchs [26] | 44 | 39 | Yes | Yes | —— | * | * | * | * | * | * |  |  | 6 |
| Kuroki [35] | 20 | 31 | Yes | Yes | —— | * | * | * | * | * | * |  |  | 6 |
| Lai [36] | 20 | 67 | Yes | Yes | —— | * | * | * | * | * | * |  |  | 6 |
| Asbun [33] | 53 | 215 | No | Yes | —— | * | * | * | * | * | * |  |  | 6 |
| Chalikonda [34] | 30 | 30 | Yes | Yes | abfg | * | * | * | * | ** | * |  |  | 7 |
| Lei [47] | 11 | 75 | unclear | Yes | —— | * | * | * | * | * | * |  |  | 6 |
| Croome [55] | 108 | 214 | Yes | Yes | —— | * | * | * | * | * | * | * | * | 8 |
| Speicher [63] | 56 | 84 | Yes | Yes | —— | * | * | * | * | * | * |  |  | 6 |
| Wang [64] | 13 | 20 | No | Yes | —— | * | * | * | * | * | * |  |  | 6 |
| Hakeem [57] | 12 | 12 | unclear | No | abcd | * | * | * | * | ** | * | * | * | 9 |
| Bao [53] | 28 | 28 | Yes | Yes | abc | * | * | * | * | * | * |  |  | 6 |
| Wellner [65] | 40 | 40 | Yes | Yes | abcdf | * | * | * | * | ** | * |  |  | 7 |
| Langan [61] | 28 | 25 | No | No | acg | * | * | * | * | * | * |  |  | 6 |
| Song [83] | 97 | 198 | No | Yes | abcf^&^ | * | * | * | * | * | * | * | * | 8 |
| Dokmak [70] | 46 | 46 | Yes | Yes | abde | * | * | * | * | ** | * |  |  | 7 |
| Mendoza [74] | 18 | 34 | unclear | Yes | —— | * | * | * | * | * | * |  |  | 6 |
| Liang [71] | 15 | 29 | unclear | Yes | —— | * | * | * | * | * | * |  |  | 6 |
| Chen [68] | 60 | 120 | Yes | Yes | abcdefg | * | * | * | * | ** | * | * | * | 9 |
| Tan [84] | 30 | 30 | No | Yes | abdef | * | * | * | * | ** | * |  |  | 7 |
| Delitto [92] | 52 | 50 | Yes | Yes | —— | * | * | * | * | * | * | * | * | 8 |
| Poves [106] | 13 | 48 | No | Yes | bd^&^ | * | * | * | * | * | * |  |  | 6 |
| Zureikat [110] | 211 | 817 | No | Yes | —— | * | * | * | * | * | * |  |  | 6 |
| Baker [88] | 22 | 49 | Yes | Yes | —— | * | * | * | * | * | * |  |  | 6 |

Factors matched between groups: a: Age; b: Sex; c: BMI; d: ASA; e: comorbidities; f: pathology; g: size; &: case matched in subgroup analysis

1. representativeness of exposed cohort; ②selection of non-exposed cohort; ③ascertainment of exposure; ④outcome not present at the start of the study; ⑤assessment of outcomes; ⑥length of follow-up; ⑦adequacy of follow-up.
