# Supplementary material for: Minimally invasive pancreaticoduodenectomy for periampullary disease: a comprehensive review of literature and meta-analysis of outcomes compared with open surgery
Source: BMC Gastroenterol. 2017 Nov 23;17:120. doi: 10.1186/s12876-017-0691-9 (PMC5701376; doi:10.1186/s12876-017-0691-9)
Supplement: Supplementary file 5 — Summary of indications and laparoscopic technical details in the meta-analysis included studies. (DOCX 26 kb) [file 12876_2017_691_MOESM5_ESM.docx]

**Additional file 5** Summary of indications and laparoscopic technical details in the meta-analysis included studies.

| **Author** | **Indications** | **Team** | **Laparoscopic technical details** | **Pancreatic anastomosis** |
| --- | --- | --- | --- | --- |
| Cho [17] | Benign or low-grade malignant periampullary disease without vascular invasion | One | LAPPPD | Extracorporeally, end-to-side PJ. |
| Zhou [30] | Periampullary cancer | One | RAPD or RAPPPD | Intracorporeally, two-layer, end-to-side PJ or PG. |
| Zureikat [31] | Periampullary disease | One | TLPD | Intracorporeally, two-layer, duct-to-mucosa, end-to-side PJ |
| Buchs [26] | Periampullary disease | One | RPD or RPPPD | Intracorporeally, use of biological glue in fragile patients for PJ or PG; PG only after RPPPD [40]. |
| Kuroki [35] | Periampullary disease without vascular invasion | One | LAPPPD | Extracorporeally, two-layer, duct-to-mucosa, end-to-side PJ. |
| Lai [36] | Periampullary tumors without vascular invasion | NR | RAPD or RAPPPD | Intracorporeally, two-layer, end-to-side PJ (duct-to-mucosa for pancreatic duct >3mm, dunking technique for pancreatic duct <3mm). |
| Asbun [33] | All pathology need PD or TP without PV invasion | Multi | TRPD, TRPPPD or TRTP | Intracorporeally, two-layer, duct-to-mucosa, end-to-side PJ with pediatric feeding tube. |
| Chalikonda [34] | Periampullary disease | Two | HLRPD | Intracorporeally, two-layer, duct-to-mucosa, end-to-side PJ with pediatric feeding tube. |
| Lei [47] | periampullary disease | One | LPD | Intracorporeally, sleeving-joint, end-to-end PJ with or without sent. |
| Croome [55] | Pancreatic ductal adenocarcinoma | NR | TLPD with or without major vascular resection | Intracorporeally, two-layer, duct-to-mucosa, end-to-side PJ with 8-cm Silastic tube [41]. |
| Speicher [63] | Periampullary disease without vein resection | One | TLPD or LAPD | NR |
| Wang [64] | Periampullary disease without vascular invasion | One | LAPD | Extracorporeally, two-layer, duct-to-mucosa, end-to-side PJ. |
| Hakeem [57] | Periampullary adenocarcinoma | One | TLPD | Intracorporeally, single loop, two-layer, duct-to-mucosa, end-to-side PJ. |
| Bao [53] | Periampullary disease without vascular invasion | NR | HLRPD or HLRPPPD | Intracorporeally, two-layer, duct-to-mucosa, end-to-side PJ with or without sent. |
| Wellner [65] | Periampullary disease | One | LAPPPD, with or without PV resection | Extracorporeally, PG, anterior and posterior gastrotomy by purse-string and full-thickness interrupted sutures. |
| Langan [61] | Periampullary adenocarcinoma | One | LAPPPD | NR |
| Song [83] | Periampullary disease | Multi | TLPPPD | Intracorporeally, PJ, two-layer, end-to-side dunking method in normal-sized ducts, or the duct-to-mucosa method in dilated pancreatic ducts. |
| Dokmak [70] | Periampullary lesions without vascular invasion | One | TLPD | Intracorporeally, one-layer, PJ with interrupted polyglactin sutures. |
| Mendoza [74] | Periampullary tumors | One | LAPPPD or LAPD | Extracorporeally, duct-to-mucosa, end-to-side PJ with polyethylene internal sent. |
| Liang [71] | Periampullary lesions without vascular invasion | One | LAPD or TLPD | Extracorporeally or intracorporeally, duct-to-mucosa, end-to-side, Blumgart-style PJ |
| Chen [68] | Resectable or borderline malignant periampullary tumors | NR | TRPD | Intracorporeally, two-layer, duct-to-mucosa, end-to-side PJ with pediatric feeding tube. |
| Tan [84] | Periampullary tumors | Two | TLPD or TLPPPD | Intracorporeally, end-to-side, mucosa-to-mucosa PJ without stent; duct-to-mucosa PJ for dilated pancreatic ducts. |
| Delitto [92] | Periampullary adenocarcinoma | NR | TLPD | Intracorporeally, end-to-end, duct-to-mucosa intussuscepting anastomosis using a running, absorbable suture without stenting. |
| Poves [106] | Periampullary tumors | One | TLPD | Extracorporeally or intracorporeally, duct-to-mucosa, end-to-side, Blumgart-style PJ or PG. |
| Zureikat [110] | Resectable pancreatic tumors without vascular invasion | Five | TRPD or TRPPPD | NR |
| Baker [88] | Periampullary disease | One | TRPPPD | Intracorporeally, two-layer, duct-to-mucosa, end-to-side PJ with pediatric feeding tube. |

NR: not report.
