# Supplementary material for: Minimally invasive pancreaticoduodenectomy for periampullary disease: a comprehensive review of literature and meta-analysis of outcomes compared with open surgery
Source: BMC Gastroenterol. 2017 Nov 23;17:120. doi: 10.1186/s12876-017-0691-9 (PMC5701376; doi:10.1186/s12876-017-0691-9)
Supplement: Supplementary file 6 — Summary of perioperative outcomes in reviewed studies with more than twenty cases of MIPD. (DOCX 37 kb) [file 12876_2017_691_MOESM6_ESM.docx]

**Additional file 6** Summary of perioperative outcomes in reviewed studies with more than twenty cases of MIPD.

| **Author** | **No.** | **MI** | **Conversion(%)** | **OP(min)** | **EBL(ml)** | **Transfusion(%)** | **LOS(days)** | **POPF(%)** | **Mortality(%)** | **LN** |
| --- | --- | --- | --- | --- | --- | --- | --- | --- | --- | --- |
| Dulucq [14] | 22 | L | 3(13.6) | 287±44 | 107 ± 48 | 3(14) | 16.2 ±2.7 | 1(4.5) | 1(4.5) | 18.4±5 |
| Palanivelu [15] | 45 | L | 0 | 370(270-640) | 65(35-395) | NR | 10.2(8-28) | 3(6.6) | 1(2.2) | 13(8-21) |
| Palanivelu [18] | 75 | L | 0 | 357(270-650) | 74(35-410) | NR | 8.2(6-42) | 5(6.7) | 1(1.3) | 14(8-22) |
| Kendrick [22] | 62 | L | 3(4.8) | 368(258-608) | 240(30-1200) | NR | 7(4-69) | 11(18) | 1(1.6) | 15(6-31) |
| Giulianotti [21] | 50 | R | 11(18.6) | 421(240-660) | 394(80-1500) | 6(12) | 22(5-85) | 19(38) | 2(4) | 18(5-45) |
| Buchs [20] | 41 | R | 2(4.9) | 435(240-660) | 389(50-1500) | 9(22) | 13(5-30) | 8(19.5) | 1(2.4) | NR |
| Zureikat [32] | 24 | L | NR | 512(327-848) | 320(50-1000) | 6(25) | 9(4-87) | 5(21) | 1(4) | NR |
| Buchs [26] | 44 | R | 2(4.5) | 444±93.5 | 387±334 | 10(22.7) | 13±7.5 | 8(18.2) | 2(4.5) | 16.8±10 |
| Zeh [39] | 50 | R | 8(16) | 568(536-629)^&^ | 350(150-625)^&^ | 11(22) | 10(8-13)^&^ | 11(22) | 1(2) | 18(13-23)^&^ |
| Kuroki [35] | 20 | L | NR | 657±191 | 377±291 | 0 | NR | 9(45) | 0 | NR |
| Lai [36] | 20 | R | 1(5) | 491.5±94 | NR | NR | 13.7±6.1 | 7(35) | 0 | 10±6 |
| Asbun [33] | 53 | L | NR | 541±88 | 195±136 | NR | 8±3.2 | 7(16.7) | 3(5.7) | 23.4±10.1 |
| Chalikonda [34] | 30 | R | 3(10) | 476 | 485 | NR | 9.8 | 4(13.3) | 1(3.3) | NR |
| Zureikat [52] | 132 | R | 11(8) | 527±103 | 300(170-600) | NR | 8(3-87) | 22(17) | 5(3.8) | 19(4-61) |
| Boggi [40] | 34 | R | 0 | 597(420-960) | 220(150-400) | 4(11.8) | 23(10-86) | 13(38.2) | 1(2.9) | 32(15-76) |
| Honda [43] | 26 | L | 2(7.7) | 519(349-778) | 322(10-1520) | 0 | NR | 6(23.1) | 0 | NR |
| Mesleh [50] | 75 | L | 10(13) | 551(390-819) | NR | NR | 7(4-68) | 7(9)* | 0 | NR |
| Gumbs [42] | 72 | R | 14(19) | 436(255-660) | 400(0-2000) | NR | 9(4-38) | 16(22) | 1(1.4) | 16(6-24) |
| Corcione [41] | 22 | L | 2(9.1) | 392(327-570) | NR | NR | 23(12-35) | 6(27) | 1(4.5) | 15(14-20) |
| Kim [45] | 100 | L | 5(5) | 475(270-810) | NR | 31(31) | 11.5(7-73) | 27(27) | 1(1) | 13(7-34) |
| Lee [46] | 42 | L | 3(7.1) | 404±31 | 375±177 | NR | 17.1±9.2 | 3(7.1)* | 1(2.3) | NR |
| Croome [55] | 108 | L | 7(6.5) | 379±94 | 492±519 | 21(19) | 6(4-118) | 12(11)* | 1(0.9) | 21.4±8.1 |
| Speicher [63] | 56 | L | NR | 415(342-487)^&^ | 421(100-700) ^&^ | NR | 10.4(7-19)^&^ | 15(26.8)* | 2(3.6) | 15(10-22)^&^ |
| Bao [53] | 28 | R | 4(14.3) | 431(340-628) | 100(50-300) | 9(32) | 7.4(5.5-17.1) | 8(29) | 2(7) | 15(8-32) |
| Wellner [65] | 40 | L | 16(40) | 343(212-510) | NR | 2(5) | 14(6-59) | 7(18)* | 1(3) | 15(7-33) |
| Langan [61] | 28 | L | 3(10.7) | 355 | 336(100-1400) | NR | 7.1 | 5(18) | 0 | NR |
| Hughes [58] | 24 | L | NR | 352(240–418) | 150(20–550) | NR | NR | 3(13) | 1(4) | NR |
| Puntambekar [62] | 38 | L | NR | 232(210-450) | 183(60-340) | NR | 14(12-25) | 3(7.9) | NR | NR |
| Song [83] | 97 | L | NR | 480±116 | 592±376 | NR | 14.1±7.7 | 29(30) | 1(1) | 15±10 |
| Paniccia [77] | 30 | L | 2(6.7) | 340(308-377) | 300 (200-400) | NR | 11(8-15) | 15(50) | 0 | 18(15–22) |
| Piedimonte [78] | 26 | B | 2(7.7) | 594(407-799) | 373(50-4000) | 6(23.1) | 8(5-57) | 9(34.6) | 1(3.8) | 22(4-56) |
| Tee [85] | 113 | L | NR | 365±111 | 345±347 | 26(23.4) | NR | 26(23)* | 5(4.4) | NR |
| Nguyen [76] | 142 | R | 11(7.7) | 529.5±103.2 | 290(150-550) | 33(23.2) | 10(7-14) | 25(17.6) | 5(3.5) | 17.5±7.8 |
| Boone [67] | 120 | R | 4(3.3) | 417±78 | 250(150-400) | 26(21.7) | 9(7-14) | 8(6.6)* | 4(3.3) | 26(19-32) |
| Dokmak [70] | 46 | L | 3(6.5) | 342(240-540) | 368(50-1200) | 5(11) | 25(6-104) | 22(48) | 1(2) | 4.7(0-32) |
| Croome [69] | 31 | L | 4(12.9) | 465±86 | 842±995 | NR | 6(4-118) | 5(16)* | 1(3.2) | 20.0±8.6 |
| Senthilnathan [80] | 130 | L | 1(0.8) | 310±34 | 110±22 | NR | 8.1±2.6 | 11(8.5)* | 2(1.5) | 18.2±4.7 |
| Chen [68] | 60 | R | 1(1.7 ) | 410±103 | 400(200-600) | 8(13.3) | 20(7.4) | 8(13.3) | 1(1.7) | 13.6±6.0 |
| Wang [86] | 31 | L | 3(9.7) | 515(465-585) | 260(150-430) | 6(19.4) | 12.6±3.7 | 8(25.8)* | 0 | 13(11-19) |
| Tan [84] | 30 | L | NR | 513.2± 56.1 | NR | 0 | 10.0±3.7 | 10(33.3) | 0 | 8.7± 1.7 |
| Liu [72] | 21 | L | 1(4.9) | 316(260-410) | 240(30-1000) | NR | NR | 1(4.9) | 0 | 14(8-26) |
| Polanco [105] | 150 | R | 11(7.3) | 515±106 | 300(150-500) | NR | NR | 26(17.3) | NR | NR |
| Coratti [90] | 36 | R | 8(22.2) | 510(390-660) | 150(30-900) | NR | 9(5-81) | 6(16.6) | 2(5.5) | NR |
| Batta [89] | 22 | R | 5(22.7) | 430(400-510) | 340(250-440) | NR | 10(6-21) | 1(4.5) | 0 | NR |
| Delitt [92] | 52 | L | 7(9) | 361±49 | 260±36 | 4(7.7) | 9±0.5 | 9(17) | 1(2) | 23±1.4 |
| Machado [102] | 50 | L | 3(6) | 436±66 | 242±144 | 12(24) | 8.4±5.1 | 13(29) | 0 | 13(6-37) |
| Wang [109] | 59 | L | 5(8.5) | 480(453-540)^&^ | 200(150-400)^&^ | 10(16.9) | 15(11-23)^&^ | 9(15.3)* | 0 | 15(9-21)^&^ |
| Zureika [110] | 211 | R | 10(4.7) | 402(257–685) | 200(30–4500) | 34(16.1) | 8(4–58) | 29(13.7)* | 4(1.9) | 27.5(7-65) |
| Kanto [96] | 828 | L | NR | NR | NR | NR | 10.2±8.5 | NR | 41(6.9) | 18.1±9.5 |
| Girgis [94] | 70 | R | 0 | 381 | 250 | 12 (17) | 9(7-14) | 9(13)* | 1(1.4) | NR |
| Liu [101] | 27 | R | 0 | 387±58 | 219±126 | NR | 17±5 | 4(14.8) | 1(3.7) | 8±5 |
| Stauffer [107] | 58 | L | 14(24.1) | 518(313-761) | 250(50-8500) | 15(25.9) | 6(4-68) | 6(11.8) | 2(3.4) | 27(9-70) |
| Baker [88] | 22 | R | 3(13.6) | 454(294-529) | 425(50-2200) | NR | 7(4-25) | 1(4.6) | 0 | NR |
| Napoli [103] | 112 | R | 3(2.7) | 526.3±102.4 | NR | NR | 22.0±2.8 | 37(33) | 4(3.6) | 47.2±12.6 |
| Cunningham [91] | 96 | R | NR | 360±70 | 187(100-350) | NR | NR | 19(19.8) | 2(2.1) | NR |
| Wang [111] | 52 | L | 0 | 276(210-498) | 80(30-300) | 0 | 12(8-16) | 2(3.8) | 0 | NR |

R: robot; L: laparoscopy; B: included both robot and laparoscopy. Note: the majority quantitative data were shown as mean ± SD or median (range); &: given as median and interquartile range (IQR); *: only include grade B and C POPF; NR: not report.
