# Supplementary material for: Minimally invasive pancreaticoduodenectomy for periampullary disease: a comprehensive review of literature and meta-analysis of outcomes compared with open surgery
Source: BMC Gastroenterol. 2017 Nov 23;17:120. doi: 10.1186/s12876-017-0691-9 (PMC5701376; doi:10.1186/s12876-017-0691-9)
Supplement: Supplementary file 7 — Summary of reasons for conversion. (DOCX 22 kb) [file 12876_2017_691_MOESM7_ESM.docx]

**Additional file 7** Summary of reasons for conversion.

| **Author** | **Reasons for conversion** |
| --- | --- |
| Zureikat [31] | failure to progress during exposure of the duodenum because of fat (n=1), bleeding from PV (n=1) |
| Buchs [26] | locally advanced tumor required difﬁcult vascular resections (n=2) |
| Lai [36] | difﬁculties in dissecting the pancreatic head and neck from the SMV/PV (n=1) |
| Asbun [33] | suspected PV involvement (n=6), failure to progress (n=3) |
| Chalikonda [34] | bleeding from the PV or its branches (n=3) |
| Croome [55] | anticipated complex venous involvement or pancreatic reconstruction (n=7) |
| Wang [64] | extensive abdominal adhesions (n=5), vascular abutment or involvement (n=4) |
| Bao [53] | difficult dissection (n=1), portal-SMV resection (n=1), bleeding (n=1) |
| Wellner [65] | peritoneal adhesion (n=1), PV adhesion (n=7), bleeding (n=2), atypical arterial anatomy (n=2), other (n=4) |
| Dokmak [70] | vascular invasion (n=1), nonprogression (n=2) |
| Chen [68] | difﬁculty in dissecting the uncinate process and requirement of PV reconstruction (n=1) |
| Delitto [92] | difﬁculties in vein resection due to preoperative underestimation of disease burden (n=5), HA injury (n=1), SMV laceration (n=1) |
| Poves [106] | slow progression/technical difﬁculty (n=3), bleeding (n=2), suspected venous invasion (n=2) |
| Baker [88] | need for vein resection or repair (n=3) |
