# Supplementary material for: Minimally invasive pancreaticoduodenectomy for periampullary disease: a comprehensive review of literature and meta-analysis of outcomes compared with open surgery
Source: BMC Gastroenterol. 2017 Nov 23;17:120. doi: 10.1186/s12876-017-0691-9 (PMC5701376; doi:10.1186/s12876-017-0691-9)
Supplement: Supplementary file 8 — Summary of perioperative outcomes in studies without quantitative data presenting mean and standard deviation. (DOCX 24 kb) [file 12876_2017_691_MOESM8_ESM.docx]

**Additional file 8** Summary of perioperative outcomes in studies without quantitative data presenting mean and standard deviation.

|  | **Operation time (min)** | | | **Blood loss (mL)** | | | **Hospital stay (days)** | | | **Retrieved lymph nodes** | | | |
| --- | --- | --- | --- | --- | --- | --- | --- | --- | --- | --- | --- | --- | --- |
|  | **MIPD** | **OPD** | ***P*** | **MIPD** | **OPD** | ***P*** | **MIPD** | **OPD** | ***P*** | **MIPD** | **OPD** | | ***P*** |
| Zureikat [31] | 456(109.5)^&^ | 372.5(117.5)^&^ | 0.01 | 300(225)^&^ | 400(750)^&^ | 0.23 | 8(8.5)^&^ | 8.5(3)^&^ | 0.71 | * | | | |
| Buchs [26] | 444(240-720)* | 559(320-850)* | <0.01 | 387(50-1500)* | 827(200-2500)* | <0.01 | 13(5-40)* | 14.6(6-47)* | 0.40 | 16.8(2-45)* | 11(2-26)* | | 0.02 |
| Lai [36] | * | | | 247(50-889) | 774.8(50-8000) | 0.03 | * | | | * | | | |
| Chalikonda [34] | 476^#^ | 366.5^#^ | <0.01 | 485^#^ | 775^#^ | 0.13 | 9.8^#^ | 13.3^#^ | 0.04 | 13.2^#^ | 11.7^#^ | | 0.25 |
| Croome [55] | * | | | * | | | 6(4-118) | 9(5-73) | <0.01 | * | | | |
| Speicher [63] | 415(342-487)^&^ | 425.5(345.8-478.8)^&^ | 0.23 | 421(100-700)^&^ | 425(300-700)^&^ | <0.01 | 10.4(7-19)^&^ | 10(8-14)^&^ | 0.13 | 15(10-22)^&^ | 12(8-16)^&^ | | 0.07 |
| Wang [64] | 594(407-779) | 553(303-892) | 0.06 | 450(100-4000) | 1000(300-6500) | 0.02 | 8(6-14) | 12(6-26) | 0.03 | 22(14-56) | 20(7-45) | | 0.09 |
| Bao [53] | 431(340-628) | 410(190-621) | 0.04 | 100(50-300)^&^ | 300(100-800)^&^ | <0.01 | 7.4(5.5-17.1)^&^ | 8.1(6.5-15.3)^&^ | 0.41 | 15 (8-32) | 19.5(11-39) | | <0.01 |
| Wellner [65] | 343(212-510) | 410(170-645) | 0.08 | NR | | | 14(6-59) | 16(10-76) | 0.06 | NR | | | |
| Langan [61] | 355^#^ | 347^#^ | 0.67 | 336(100-1400) | 454(100-1200) | 0.08 | 7.1^#^ | 9.4^#^ | 0.02 | NR | | | |
| Dokmak [70] | 342(240-540) | 264(120-400) | <0.01 | 368(50-1200) | 293(50-1200) | 0.16 | 25(6-104) | 23(7-115) | 0.59 | 20(8-59) | 25(8-47) | | 0.80 |
| Mendoza [74] | 530(420-610)^&^ | 357(295-690)^&^ | <0.01 | 500(300-800)^&^ | 450(100-1100)^&^ | 0.10 | 13(7-18)^&^ | 15.5(11-36)^&^ | <0.01 | * | | | |
| Liang [71] | 342 (290–507) | 358 (270–626) | 0.99 | NR | | | 8(4-44) | 9(4-104) | 0.61 | 9 (5–22) | 14 (0–40) | | 0.09 |
| Chen [68] | * | | | 400(200-600) | 500(350-800 | <0.01 | * | | | * | | | |
| Poves [106] | NR | | | NR | | | 14(7.5-15.5) | 15.5(8-21.8) | 0.36 | NR | | | |
| Zureikat [110] | 402(257-685) | 300(107-840) | <0.01 | 200(30-4500) | 300(20-7350) | <0.01 | 8(4-58) | 8(4-148) | 0.98 | 27.5(7-65) | 19(3-72) | <0.01 | |
| Baker [88] | 454(294-529) | 364(213-948) | 0.04 | 425(50-2200) | 650(150-6100) | 0.04 | 7(4-25) | 9(5-48) | NS | NR | | | |

Note: the majority were given as median and range; &: given as median and interquartile range (IQR); #: only given median; *: reported mean and standard deviation in original article; NR: not report; NS not signiﬁcant.
