# Supplementary material for: Minimally invasive pancreaticoduodenectomy for periampullary disease: a comprehensive review of literature and meta-analysis of outcomes compared with open surgery
Source: BMC Gastroenterol. 2017 Nov 23;17:120. doi: 10.1186/s12876-017-0691-9 (PMC5701376; doi:10.1186/s12876-017-0691-9)
Supplement: Supplementary file 9 — Summary of the specific reoperation reasons. (DOCX 22 kb) [file 12876_2017_691_MOESM9_ESM.docx]

**Additional file 9** Summary of the speciﬁc reoperation reasons.

| **Author** | **Group** | **Reoperation cases** |
| --- | --- | --- |
| Zhou [30] | OPD | abdominal abscess due to PF (n=1) |
| Zureikat [31] | MIPD | gastric staple line bleeding (n=1) |
| Buchs [26] | MIPD | grade C PF (n=1), GDA bleeding (n=1) |
|  | OPD | grade C PF (n=2), HA bleeding (n=1), biliocutaneous ﬁstula (n=1), wound infection (n=1) |
| Lai [36] | MIPD | HA pseudoaneurysm (n=1), colon ischemia (n=1) |
|  | OPD | grade C PF (n=1), other (n=2) |
| Chalikonda [34] | MIPD | grade C PF (n=1), GDA bleeding (n=1), wound dehiscence |
|  | OPD | grade C PF (n=3), hepatojejunostomy dehiscence (n=2), omental bleeding (n=1), biliary ﬁstula (n=1) |
| Wellner [65] | MIPD | bleeding at PG (n=5), pancreatitis (n=4), abdominal abscess due to PF (n=3), adhesion ileus (n=2), burst abdomen (n=1), hepatoenterostomy leak (n=1), colon ischemia (n=1) |
|  | OPD |  |
| Dokmak [70] | MIPD | bleeding and collections (n=9), gastrojejunostomy leak (n=1), small bowel incarceration (n=1) |
| Liang [71] | MIPD | grade C PF (n=3) |
|  | OPD | grade C PF (n=1) |
| Chen [68] | MIPD | afferent loop obstruction (n=2) |
|  | OPD | bleeding due to grade C PF (n=4) |
