# Supplementary material for: Minimally invasive pancreaticoduodenectomy for periampullary disease: a comprehensive review of literature and meta-analysis of outcomes compared with open surgery
Source: BMC Gastroenterol. 2017 Nov 23;17:120. doi: 10.1186/s12876-017-0691-9 (PMC5701376; doi:10.1186/s12876-017-0691-9)
Supplement: Supplementary file 10 — Summary of the specific perioperative death reasons. (DOCX 23 kb) [file 12876_2017_691_MOESM10_ESM.docx]

**Additional file 10** Summary of the speciﬁc perioperative death reasons.

| **Author** | **Group** | **Death cases** |
| --- | --- | --- |
| Zhou [30] | OPD | abdominal hemorrhage secondary to PF (n=1) |
| Zureikat [31] | MIPD | multisystem organ failure secondary to sepsis (n=1) |
| Buchs [26] | MIPD | cardiac arrhythmia (n=1), GI bleeding (n=1) |
|  | OPD | cardiac arrest (n=1) |
| Asbun [33] | MIPD | myocardial infarction(n=1), sepsis (n=1), GI bleeding (n=1) |
| Chalikonda [34] | MIPD | PV bleeding (n=1) |
| Wang [64] | MIPD | refractory sepsis (n=1) |
|  | OPD | acute hepatic and renal failure (n=1), myocardial infarction (n=1), PV bleeding (n=1), abdominal sepsis (n=1) |
| Hakeem [57] | OPD | severe sepsis (n=1) |
| Bao [53] | MIPD | myocardial infarction and aspiration (n=1), stroke (n=1) |
|  | OPD | sepsis from grade C PF (n=1), ventricular fibrillation arrest (n=1) |
| Wellner [65] | MIPD | septicn multiorgan failure due to necrotizing colitis (n=1) |
| Dokmak [70] | MIPD | PF, bleeding and early liver metastases (n=1) |
| Liang [71] | MIPD | PF and pulmonary embolism (n=1) |
|  | OPD | liver failure due to hepatic ﬁbrosis (n=1) |
| Chen [68] | MIPD | renal failure (n=1) |
|  | OPD | DIC (n=1), massive bleeding (n=1), sepsis (n=1) |
| Tan [84] | OPD | bleeding and infection due to grade C PF (n=1) |
| Delitto [92] | MIPD | post-pancreatectomy hemorrhage (n=1) |
| Poves [106] | OPD | PF (n=1), pancreatectomy hemorrhage (n=1) |
| Baker [88] | OPD | PF (n=1), respiratory failure (n=1) |
